# Ecological and genetic diversity of plant growth-promoting genes in rhizobacteria isolated from the rhizosphere of wild flora on Mount Erciyes, Türkiye

**DOI:** 10.3389/fpls.2025.1657785

**Published:** 2025-09-03

**Authors:** Ugur Azizoglu

**Affiliations:** ^1^ Department of Crop and Animal Production, Safiye Cikrikcioglu Vocational College, Kayseri University, Kayseri, Türkiye; ^2^ Genome and Stem Cell Research Center, Erciyes University, Kayseri, Türkiye

**Keywords:** plant hormones, gene analyses, biodiversity, rhizobacteria, PGPR

## Abstract

**Introduction:**

This study is the first to investigate the genetic diversity of plant growth-promoting genes in rhizobacteria isolated from the wild ecology of Mount Erciyes, Türkiye. It has a flora rich in flowering plants, with 1170 plant taxa, 194 of which are endemic to the area.

**Methods:**

A total of 165 bacterial isolates, including *Azotobacter* sp., *Azospirillum* sp., and *Bacillus* sp., were screened for genes associated with plant growth promotion: nitrogen fixation (*nif*), indole pyruvate decarboxylase (*ipdC*), 1-aminocyclopropane-1-carboxylate deaminase (*accd*), phosphate-solubilizing genes (*Acpho*, *Alpho* and *phy*), and siderophore biosynthesis (*sd*).

**Results:**

The analysis revealed significant genetic variability across isolates, particularly for *nif* and *sd* genes, with distinct band patterns indicating genetic diversity among *Azospirillum* and *Bacillus* isolates.

**Discussion:**

The findings emphasize the role of these rhizobacteria in nutrient cycling and stress resilience, potentially enhancing plant growth in nutrient-limited soils. In the current study, it contributes to understanding microbial biodiversity in Mount Erciyes and suggests a promising potential for sustainable agriculture through plant-microbe interactions.

## 1 Introduction

Recent advancements in agricultural research have increasingly focused on sustainable practices to enhance crop productivity while minimizing environmental impacts. In semi-arid regions like the southeastern part of the Central Anatolian Plateau, including Kayseri, Türkiye, cereal and legume cultivation faces challenges due to nutrient-poor aridisols and limited water availability. Plant growth-promoting rhizobacteria (PGPR) have emerged as a promising solution to these challenges, offering eco-friendly alternatives to chemical fertilizers. Studies have demonstrated that PGPR strains, such as *Azospirillum* and *Bacillus*, can significantly enhance crop yields. For instance, a study by Çakmakçı et al. (2017) reported that *Azospirillum* brasilense and *Bacillus subtilis* increased wheat grain yields by up to 30% in semi-arid conditions through mechanisms like nitrogen fixation and phosphate solubilization. Similarly, Erturk et al. (2012) found that co-inoculation of *Bacillus* and *Rhizobium* improved chickpea nodulation and yield by 25% in nutrient-deficient soils, highlighting the potential of PGPR for sustainable agriculture in regions like Kayseri.

The application of PGPR extends beyond yield enhancement to include stress tolerance and soil health improvement. Research by Swarnalakshmi et al. (2020) emphasized that PGPR, including *Azospirillum* and *Bacillus*, promote legume growth by producing phytohormones like indole-3-acetic acid (IAA) and solubilizing phosphorus, reducing reliance on synthetic fertilizers. Additionally, PGPR contribute to biocontrol by producing siderophores and antimicrobial compounds, as noted in a meta-analysis by Zeffa et al. (2020), which reported an 11.4% increase in soybean nodulation with *Bradyrhizobium* and *Bacillus* co-inoculation. These findings underscore the multifaceted roles of PGPR in enhancing nutrient cycling and plant resilience, critical for sustainable farming in semi-arid ecosystems.

Türkiye’s diverse ecological landscape, particularly around Mount Erciyes in Kayseri, supports a rich biodiversity that remains underexplored for microbial resources. The southeastern part of the Central Anatolian Plateau, including the Kayseri region, is dominated by cereal and legume cultivation on semi-arid, nutrient-poor aridisols. Plant growth-promoting rhizobacteria (PGPR) strains such as *Azospirillum* and *Bacillus* have been shown to increase wheat and barley grain yields by up to 40%, providing a sustainable, low-input alternative to chemical fertilizers that could greatly benefit local farmers (Barış et al., 2014). Türkiye’s diverse ecological structure fosters high levels of endemism and genetic diversity. Mount Erciyes, a prominent stratovolcano situated approximately 25 km southwest of Kayseri, rises from the plains of Sultansazlığı and hosts a flora rich in flowering plant species. Of the 1170 plant taxa identified in this region, 194 are endemic (Vural and Aytaç, 2005). Although many studies have explored the biodiversity of plant and animal life on Mount Erciyes, a significant knowledge gap remains regarding the rhizobacteria inhabiting this unique environment. To date, there has been no research on PGPR or their associated functional genes in this area. PGPR are beneficial bacteria commonly isolated from soil and are known to promote plant growth through various mechanisms. These bacteria belong to diverse genera, including *Alcaligenes*, *Agrobacterium*, *Azospirillum*, *Azotobacter*, *Arthrobacter*, *Bacillus*, *Bradyrhizobium*, *Burkholderia*, *Caulobacter*, *Chromobacterium*, *Enterobacter*, *Erwinia*, *Flavobacterium*, *Herbaspirillum*, *Klebsiella*, *Mesorhizobium*, *Micrococcus*, *Pseudomonas*, *Rhizobium*, *Rhodococcus*, and *Serratia* (Egamberdiyeva, 2005). Among these genera, *Azospirillum* and *Azotobacter* are noteworthy for their nitrogen-fixing abilities, while *Bacillus* is notable for producing plant hormones, siderophores, and phosphate-solubilizing enzymes.

In this study, we focused on characterizing the genetic diversity of plant growth-promoting genes within rhizobacterial populations, specifically targeting *Azospirillum* sp., *Azotobacter* sp., and *Bacillus* sp., isolated from the unique flora of Mount Erciyes. To assess their plant growth-promoting potential, we screened for genes associated with key PGPR functions. These included nitrogen fixation genes (*nif*), indole pyruvate decarboxylase (*ipdC*) involved in auxin (IAA) biosynthesis, 1-aminocyclopropane-1-carboxylate deaminase (*accd*) associated with ethylene modulation, and genes involved in phosphate solubilization, such as acid phosphatase (*Acpho*), alkaline phosphatase (*Alpho*), and phytase (*phy*). Additionally, we screened for siderophore biosynthesis genes (*sd*) essential for iron acquisition and plant growth enhancement.

## 2 Materials and methods

### 2.1 Collection of soil samples

Soil samples were collected from various soil types (clayey, sandy, and loamy soils) within the vegetative regions of Mount Erciyes, with each sample consisting of a 1 kg mixture obtained by combining 100 g of soil from 10 different spots within an area. The sampling sites were strategically distributed to capture a range of ecological niches, including areas near endemic plant species and agricultural fields, to ensure a comprehensive representation of the rhizobacterial populations associated with the region’s unique flora. Samples were taken from the plant root-soil interface area (5-10 cm depth) in locations free from commercial microbial fertilizers. In total, 22 soil samples were collected, with GPS coordinates recorded for each sampling location (**Table 1**).

**Table 1:** Soil samples and their GPS locations.

Location name	Latitude	Longitude	Altitude (m)
1. Kıranardı	N 38 36’ 51,90640”	E 35 32’ 10,47160”	1616
2. Hisarcık	N 38 37’ 32,01250”	E 35 30’ 25,42830	1542
3. Endürlük	N 38 38’ 9,70420”	E 35 33’ 4,56050”	1376
4. Kepez	N 38 36’ 59,83560”	E 35 39’ 55,80450”	1697
5. Yazyurdu	N 38 34’ 48,22060”	E 35 40’ 40,70250”	1641
6. Cebir	N 38 34’ 5,21330”	E 35 37’ 0,67020	1942
7. Koçcağız	N 38 30’ 17,01790”	E 35 42’ 14,21860”	1603
8. Çaylıca	N 38 24’ 57,44160”	E 35 37’ 11,02440”	1475
9. Soysallı	N 38 23’ 24,73720”	E 35 21’ 43,81860”	1074
10. Çayırözü	N 38 24’ 58,48490”	E 35 17’ 20,58580”	1073
11. Kulpak	N 38 27’ 27,01850”	E 35 19’ 40,23290”	1287
12. Ahmet pınarı	N 38 27’ 49,09050”	E 35 24’ 7,49670”	1863
13. Tekir	N 38 30’ 45,68570”	E 35 31’ 15,93220”	2209
14. Hacılar	N 38 35’ 26,66210”	E 35 27’ 54,21670”	2074
15. Sakar Çiftliği	N 38 37’ 29,16490”	E 35 23’ 52,13680”	1461
16. Kızılören	N 38 36’ 6,01990”	E 35 20’ 21,60570”	1542
17. Sarıkürklü	N 38 39’ 5,77110”	E 35 18’ 8,17520”	1133
18. Subaşı	N 38 33’ 6,09570”	E 35 14’ 13,52140”	1141
19. Kızılini	N 38 32’ 7,63710”	E 35 17’ 28,36790”	1623
20. Şehşaban	N 38 28’ 52,61930”	E 35 14’ 48,15300”	1156
21. Karpuzsekisi	N 38 41’ 4,99640”	E 35 18’ 50,90630”	1031
22. Hürmetçi	N 38 42’ 0,09030”	E 35 18’ 46,35280”	1031

### 2.2 Physicochemical characterization of soil samples

A total of 22 different soil samples were analyzed for texture, pH (potential of hydrogen), lime (calcium carbonate equivalent, CaCO_3_), organic matter (OM), electrical conductivity (EC), phosphorus (P), total nitrogen (N), iron (Fe), copper (Cu), zinc (Zn), and manganese (Mn) properties. Soil texture was determined using the Bouyoucos hydrometer method (Bauder, 2000). Soil pH was measured potentiometrically with a glass electrode pH meter (Cole-Parmer Jenway 3510 Standard Digital pH Meter) using a 1:2.5 soil-to-water ratio (Mclean, 1982). Lime content was determined volumetrically with a Scheibler calcimeter. Organic matter content was measured using the Walkley–Black wet oxidation method (Walkley and Black, 1934). Exchangeable cations (Na^+^, Ca²^+^, Mg²^+^, and K^+^) were extracted by shaking the soils with 1 N ammonium acetate (pH 7.0) and quantified by inductively coupled plasma optical emission spectrometry (ICP-OES) (Rhoades, 1982). Available phosphorus was determined using the molybdenum blue colorimetric method by measuring the absorbance of the resulting blue solution at 660 nm with a spectrophotometer (Olsen and Sommers, 1982). Total nitrogen content was calculated using the micro-Kjeldahl method after wet digestion with a mixture of salicylic acid, sulfuric acid, and salt. Micronutrients, including Fe, Cu, Zn, and Mn, were extracted using the DTPA (diethylenetriaminepentaacetic acid) method and quantified by ICP-OES (Lindsay and Norvell, 1978).

### 2.3 Bacteria isolation and culture conditions

#### 2.3.1 Isolation and identification of *Azotobacter* sp.

To isolate *Azotobacter* sp. from soil samples, 10 grams of each soil sample were mixed with 90 ml of dH_2_O. After thorough vortex mixing, the samples were left to settle at room temperature for approximately 20 min. Following this, 1 ml of the supernatant was transferred to 50 ml flasks containing Ashby liquid medium (pH 7.3) (per liter: 20 g mannitol, 0.2 g K_2_HPO_4_, 0.2 g MgSO_4_•7H_2_O, 0.2 g NaCl, 0.1 g K_2_SO_4_, 5 g CaCO_3_, and 1 ml of a microelement solution containing: 1 g H_3_BO_3_, 2 g FeSO_4_ **•**7H_2_O, 1 g MnCl_2_ **•**4H_2_O, 1 g Na_2_MoO_4_•2H_2_O, 0.5 g NaBr, and 0.2 g ZnSO_4_•7H_2_O) and incubated at 28°C for 5-7 days at 200 rpm in a shaking incubator (Bakulin et al., 2007). From the resulting culture, 1 ml was taken and serially diluted (10^-4^ to 10^-7^) before being plated on Ashby agar (*Azotobacter* Agar with Mannitol). Plates were incubated at 28°C for 5-7 days. *Azotobacter* colonies grown on the selective medium were stored at +4°C until DNA isolation (Bakulin et al., 2007).

#### 2.3.2 Isolation and identification of *Azospirillum* sp.

To isolate *Azospirillum* sp., 10 grams of each soil sample were mixed with 90 ml of sterile distilled water. After thorough vortex mixing, the samples were left to settle at room temperature for approximately 20 minutes. Then, 1 ml of the liquid portion was serially diluted (10^-^² to 10^-7^) (Baldani and Reis, 2014), and added to 10 ml of N-free medium (liquid NFb medium, 1 L, pH 6.5: 5 g malic acid, 0.05 g yeast extract, 0.5 g K_2_HPO_4_, 0.2 g MgSO_4_ **•**7H_2_O, 0.1 g NaCl, 0.02 g CaCl_2_ **•**2H_2_O, 0.04 g CuSO_4_ **•**5H_2_O, 0.12 g ZnSO_4_ **•**7H_2_O, 1.4 g H_3_BO_3_, 1 g Na_2_MoO_4_ **•**2H_2_O, 1.175 g MnSO_4_ **•**H_2_O, 2 ml bromothymol blue [5 g/L in 0.2 N KOH], 2 ml Fe-EDTA solution [16.4 g/L], 4 ml vitamin solution [10 mg biotin; 20 mg pyridoxal-HCl, pH adjusted with KOH; for solid NFb medium, add 15 g agar]) (Baldani and Reis, 2014). *Azospirillum* sp. colonies grown on the selective medium were stored at +4°C until DNA isolation.

#### 2.3.3 Isolation and identification of *Bacillus* sp.

To isolate *Bacillus* sp., 10 grams of each soil sample were mixed with 90 ml of sterile distilled water. After thorough vortexing, the samples were allowed to settle at room temperature for approximately 5 minutes. Then, 1 ml of the supernatant was transferred to 50 ml flasks containing 10 ml of Luria-Bertani (LB) Broth medium buffered with 0.25 M sodium acetate. The flasks were incubated in a shaking incubator at 30°C for 4 hours at 200 rpm. Following incubation, 1 ml of each sample was placed in sterile eppendorf tubes and heat-treated at 80°C for 5-10 minutes. Afterward, samples were plated on LB agar plates and incubated overnight at 30°C (Travers et al., 1987; Katı et al., 2016). *Bacillus* bacterial colonies grown on the selective medium were stored at +4°C until DNA isolation.

### 2.4 DNA isolation and amplification of plant-growth-promoting genes

DNA isolation was performed using the Bio Basic Bacterial Genomic DNA Isolation Kit (BS624), following the manufacturer’s recommended protocol. From each isolated DNA sample, 5 μl were loaded onto a 1% (w/v) agarose gel prepared in 1x TAE buffer (Tris-Acetic Acid-EDTA, pH 8.0) containing 10 μl ethidium bromide, and electrophoresed at 80 V for 1 hour. DNA presence was verified using a Biorad gel imaging system.

To determine whether the rhizobacteria identified as *Azotobacter*, *Azospirillum*, and *Bacillus* carried genes promoting plant growth, PCR analysis was conducted using primers (*nifH-1, nifH-2, nifH-3, nifH-4, nifH-5, nifH-6, nifH-7, nifH-8, nifH-9*, *Acpho-1, Acpho-2, Acpho-3, Acpho-4*, *Alpho-1, Alpho-2, Alpho-3, phy-1, phy-2, sd-1, sd-2, sd-3, accd*, *ipdC-1, ipdC-2, ipdC-3, ipdC-4, ipdC-5, ipdC-6, ipdC-7, ipdC-8* and *ipdC-9)* for the *nif*, *ipdC*, *accd*, *Acpho*, *Alpho*, *phy*, and *sd* genes (**Table 2**) (Helmut et al., 2004; Ding et al., 2005; Wilson et al., 2006; Sakurai et al., 2008; Raddadi et al., 2008; Bawane et al., 2011; Shime-Hattori et al., 2011; Susilowati and Setyowati, 2016; Ahmed et al., 2017; Kirillov et al., 2017; Gaiero et al., 2018; Meng et al., 2019).

**Table 2:** Primer pairs used to identify growth-promoting genes by PCR analysis.

Primers	Primer sequence (5´→3´)		Annealing (°C)	Target gene
***nifH-1***	F	CAGACACGAAGAAGCCGGGC	50	Nitrogenase
	R	GACCAGCAGCTTGTTGTTGA		
***nifH-2***	F	CGCCGGCGCAGTGTTTGCGG	50	
	R	CACTCGTTGCAGCTGTCGGC		
***nifH-3***	F	GGTTGTGACCCGAAAGCTGA	50	
	R	GCGTACATGGCCATCATCTC		
***nifH-4***	F	GGCTGCGATCCCGAAAGGCCGACTTCCGAACCCG	55	
	R	CTGGCAGCCTTGTTCTTCGCGGATCGGGCATGGC		
***nifH*-5**	F	GGCTGCGATCCAAGGCCGATCACCCG	50	
	R	CTGGCCTTGTTTCGCGGATGGCATGGC		
***nifH-6***	F	GGCAAGGGCGGTATCGGCAAGTC	55	
	R	CCATCGTGATCGGGTCGGGATG		
***nifH-7***	F	GACCCGCCTGATCCTGCACG	55	
	R	GTTCTCTTCCAGGAAGTTGATCGA		
***nifH-8***	F	GACCCGCCTGATCCTGCACG	55	
	R	GTCGTAGGCGCCGTTC		
***nifH-9***	F	GACCCGCCTGATCCTGCACG	55	
	R	CATGACGATGTAGATTTCCTGG		
***Acpho-1***	F	AAGAGGGGCATTACCACTTTATTA	55	Acid phosphatase
	R	CGCCTTCCCAATCRCCATACAT		
***Acpho-2***	F	CGGCTCCTATCCGTCCGG	58	
	R	CAACATCGCTTTGCCAGTG		
***Acpho-3***	F	GTCGGCTTTGATATCGATGA	55	
	R	CGAGCGACCTCTTTTGGAAT		
***Acpho-4***	F	TCTTCGGTGATAACTTAGGTGACTT	55	
	R	AATCGTATAAAGCGCCTTCC		
***Alpho-1***	F	CAGTGGGACGACCACGAGGT	57	Alkaline phosphatase
	R	GAGGCCGATCGGCATGTCG		
***Alpho-2***	F	GCCGGAGCTCATGAGTTTATTTAAACAGGTTCGAT	50	
	R	GCCGCTGCAGTTATTTGCCGCTTTTTAAGATG		
***Alpho-3***	F	GGAATTCCATATGGGTTTCTTACGCAACAGAAT	55	
	R	ATAGTTTAGCGGCCGCTCTGGCGTATTTTTTGAATAGCT		
***phy-1***	F	TATGATTTTCCGTTGAAC	48	Phytase
	R	ATTCCGTCTGTATCGCTTGT		
***phy-2***	F	CTGTCTGATCCTTATCATTT	52	
	R	TCCGCTTCTGTCGGTCA		
***sd-1***	F	GAGAATGGATTACAGAGGAT	55	Siderophore biosynthesis
	R	TTATGAACGAACAGCCACTT		
***sd-2***	F	GGAGAATGGATTACAGAGG	48	
	R	GTCGTCATATAAATTTCCAG		
***sd-3***	F	ACGATTGCACAATATGAAAGA	55	
	R	CAATGGTTTGGAACTTCATG		
***accd***	F	GTGAACCACCTGAATGTA	50	acc deaminase
	R	AAACGAGATGATTTACTTGG		
***ipdC-1***	F	CACTTGAAAACGCAATATACTG	50	Indole pyruvate decarboxylase
	R	AAGAATTTGCTGGCCGAATCT		
***ipdC-2***	F	CATTTGAAAACTCACTATACTG	51	
	R	AAGAATTTGTATGCCGAATCT		
***ipdC-3***	F	CACTTGAAAACTCACTATACTG	53	
	R	AAGAATTTGCATGCCGAATCT		
***ipdC-4***	F	CATTTGAAAACGCAATATACTG	52	
	R	AAGAATTTGTTGGCCGAATCT		
***ipdC-5***	F	CACTTGAAAACTCAATATACTG	50	
	R	AAGAATTTGCTTGCCGAATCT		
***ipdC-6***	F	CACTTGAAAACGCACTATACTG	55	
	R	AAGAATTTGCTGGCCGAATCT		
***ipdC-7***	F	CACTTGAAAACGCAATATACTG	54	
	R	AAGAATTTGTTGGCCGAATCT		
***ipdC-8***	F	CACTTGAAAACGCAATATACTG	54	
	R	AAGAATTTGTAGGCCGAATCT		
***ipdC-9***	F	CACTTGAAAACGCAATATACTG	50	
	R	AAGAATTTGTATGCCGAATCT		

Each reaction contained the reagents at a final concentration as 2.4 mM MgCl_2_, 1× taq buffer, 0.25 mM dNTPmix, 0.3 pmol primers (each), 0.5 U taq DNA polymerase, and 30–100 ng of template DNA. The PCR amplification was performed under the following conditions: Initial denaturation at 95°C for 5 min, followed by 30 cycles at 94°C for 1 min, Tm C for 1 min, 72°C for 2 min, and a final extension step at 72°C for 10 min (Raddadi et al., 2008). Following PCR, 5 μl of each product was loaded onto a 1% (w/v) agarose gel prepared in 1x TAE buffer (Tris-Acetic Acid-EDTA, pH 8.0) containing 10 μl ethidium bromide, and electrophoresed at 80 V for 1 hour. DNA bands were visualized on a Biorad gel imaging system. GeneRuler 100 bp Plus DNA Ladder (Thermo Scientific), ranging from 100 to 3000 bp, was used as the molecular size marker in all gels to estimate the fragment sizes.

### 2.5 Statistical analysis

Statistical analyses were performed using the MSTAT-C statistical software (Michigan State University, USA). A one-way analysis of variance (ANOVA) was applied to assess differences in soil physicochemical properties among the 22 sampling sites on Mount Erciyes. Mean comparisons were conducted using the Tukey-Kramer Honestly Significant Difference (HSD) *post hoc* test at a 99% confidence level (p < 0.01). All parameters presented in **Table 3**, including pH, electrical conductivity (EC), organic matter (OM), nitrogen (N), lime content, phosphorus (P), calcium (Ca), potassium (K), magnesium (Mg), sodium (Na), copper (Cu), iron (Fe), manganese (Mn), and zinc (Zn), were subjected to this analysis. However, due to the absence of replicate measurements per location (n=1), within-group variance could not be estimated, rendering traditional significance testing inconclusive. Consequently, the data were interpreted descriptively, focusing on ranges and patterns across locations.

**Table 3:** Main physico-chemical characteristics of the soil from Mount Erciyes.

Locations	Texture	pH	EC µs/cm	OM %	N %	Lime %	P kg/da	Ca mg/kg	K mg/kg	Mg mg/kg	Na mg/kg	Cu mg/kg	Fe mg/kg	Mn mg/kg	Zn mg/kg
1. Kıranardı	Clay loam	7.36	206.0	2.91	0.15	1.53	1.17	739.6	135.0	69.5	61.8	0.52	0.45	0.72	0.65
2. Hisarcık	Clay loam	7.76	365.0	2.45	0.12	1.23	2.23	788.5	143.0	43.0	74.5	0.62	0.62	0.75	0.78
3. Endürlük	Clay loam	7.42	272.0	2.63	0.13	2.65	3.45	1256.3	132.5	37.5	64.5	0.54	0.69	0.81	0.81
4. Kepez	Clay loam	7.38	212.0	2.51	0.13	3.44	1.56	1365.2	122.5	56.0	78.8	0.51	0.57	0.86	0.95
5. Yazyurdu	Clay loam	7.30	220.0	2.02	0.10	3.21	2.58	1452.2	131.5	57.5	101.2	0.45	0.69	0.91	0.91
6. Cebir	Clay loam	7.16	174.0	2.45	0.12	5.42	3.45	1574.5	139.0	43.0	123.3	0.63	0.75	0.56	0.88
7. Koçcağız	Loam	6.99	222.0	3.12	0.16	3.55	10.25	1956.0	120.5	39.0	86.5	0.58	0.78	0.85	1.02
8. Çaylıca	Loam	6.97	486.0	2.75	0.14	6.85	8.54	1845.0	128.0	29.5	95.6	0.51	0.59	0.75	1.11
9. Soysallı	Clay loam	8.10	1709.0	1.85	0.09	7.41	9.65	1923.0	127.5	32.5	1523.6	0.54	1.02	0.46	1.06
10. Çayırözü	Clay loam	8.27	1708.0	5.02	0.25	18.42	17.22	2027.0	180.5	107.0	1648.1	0.43	0.56	0.84	1.15
11. Kulpak	Clay loam	8.13	622.0	4.88	0.24	15.25	15.24	1856.3	162.0	87.5	755.2	0.42	0.68	0.75	0.78
12.Ahmet pınarı	Clay loam	7.64	234.0	5.12	0.26	12.35	10.26	2063.0	163.0	84.0	302.5	0.96	0.95	0.56	0.89
13. Tekir	Loam	6.54	168.4	4.56	0.23	11.00	12.47	2024.0	167.5	62.5	123.6	0.58	0.84	0.91	1.25
14. Hacılar	Clay loam	7.14	186.5	4.75	0.24	12.36	8.65	2156.0	156.0	68.0	154.8	0.65	0.71	0.88	1.14
15.Sakar Çiftliği	Clay loam	7.40	236.0	3.86	0.19	8.45	9.36	1863.0	144.0	72.5	112.3	0.43	0.80	0.76	0.78
16. Kızılören	Clay loam	7.22	152.1	3.95	0.20	9.12	11.25	2036.0	122.5	42.5	163.9	0.57	0.75	0.94	0.94
17. Sarıkürklü	Clay loam	7.51	145.1	4.12	0.21	9.76	8.54	1765.0	143.0	56.0	102.5	0.61	0.62	0.58	0.91
18. Subaşı	Clay loam	7.55	337.0	4.44	0.22	5.45	6.32	1742.0	156.0	34.5	185.2	0.25	0.69	0.91	0.66
19. Kızılini	Clay loam	7.33	116.0	4.45	0.22	4.66	5.48	1556.3	182.5	39.0	223.6	0.36	0.88	0.78	0.78
20. Şehşaban	Clay loam	7.63	295.0	3.80	0.19	3.53	3.58	968.5	132.0	31.5	148.8	0.41	0.57	0.52	1.02
21. Karpuzsekisi	Clay loam	7.78	424.0	3.95	0.20	5.45	6.53	1623.5	144.0	43.0	123.6	0.32	0.69	0.63	1.15
22. Hürmetçi	Clay loam	8.08	4760.0	4.12	0.21	6.12	4.77	1552.3	146.0	38.5	4565.2	0.25	0.72	0.59	1.03

No significant differences were observed among the locations for the measured physicochemical parameters, as replicate measurements were unavailable for statistical testing (p-values undefined; one-way ANOVA and Tukey-Kramer HSD test at p < 0.01). Data are presented descriptively to highlight variations in ranges.

## 3 Results

### 3.1 Physicochemical characterization of soil samples

**Table 3** summarizes the main physicochemical properties of the soil samples collected from the sampling sites. Soil samples collected from 22 distinct locations within the vegetative regions of Mount Erciyes were subjected to comprehensive physicochemical characterization to assess their properties and suitability for supporting rhizobacterial populations. These locations, spanning diverse microenvironments across the slopes of Mount Erciyes, were selected to capture variations in soil texture, nutrient content, and ecological niches. **Table 3** summarizes the primary physicochemical properties of the soil samples, including texture, pH, electrical conductivity (EC), organic matter (OM) content, total nitrogen (N), lime content, and concentrations of key nutrients such as phosphorus (P), calcium (Ca), potassium (K), magnesium (Mg), sodium (Na), copper (Cu), iron (Fe), manganese (Mn), and zinc (Zn).

Soil texture analysis revealed that the majority of the samples (19 out of 22) were classified as clay loam, while three locations (Koçcağız, Çaylıca, and Tekir) exhibited a loamy texture. This predominance of clay loam soils suggests a high capacity for nutrient and water retention, which is critical for supporting microbial activity and plant growth in the semi-arid conditions of the Central Anatolian Plateau. The pH values ranged from slightly acidic to moderately alkaline (6.54 to 8.27), with most samples falling within a neutral to slightly alkaline range (7.14–8.13). Notably, the Tekir location exhibited the most acidic pH (6.54), while Çayırözü had the highest pH (8.27), potentially influencing the solubility and availability of nutrients and the activity of rhizobacteria.

Electrical conductivity (EC), an indicator of soil salinity, varied significantly across the sampling sites, ranging from 116.0 µS/cm (Kızılini) to 4760.0 µS/cm (Hürmetçi). The exceptionally high EC at Hürmetçi suggests potential salinity stress, which could impact microbial communities and plant growth. Organic matter (OM) content ranged from 1.85% (Soysallı) to 5.12% (Ahmet Pınarı), indicating moderate to high organic content that supports microbial activity. Total nitrogen (N) content followed a similar trend, ranging from 0.09% (Soysallı) to 0.26% (Ahmet Pınarı), reflecting the variability in soil fertility across the sites.

Lime content, which affects soil structure and nutrient availability, was notably high at Çayırözü (18.42%) and Kulpak (15.25%), potentially influencing pH and microbial processes.

Nutrient analysis revealed variation in phosphorus (P), calcium (Ca), potassium (K), magnesium (Mg), and sodium (Na) concentrations. Phosphorus levels ranged from 1.17 kg/da (Kıranardı) to 17.22 kg/da (Çayırözü), indicating a wide range of phosphate availability that could influence the activity of phosphate-solubilizing rhizobacteria. Calcium concentrations were consistently high, ranging from 739.6 mg/kg (Kıranardı) to 2156.0 mg/kg (Hacılar), reflecting the calcareous nature of the region’s soils. Potassium and magnesium levels showed moderate variation, with K ranging from 120.5 mg/kg (Koçcağız) to 182.5 mg/kg (Kızılini) and Mg from 29.5 mg/kg (Çaylıca) to 107.0 mg/kg (Çayırözü). Sodium levels were notably elevated at Hürmetçi (4565.2 mg/kg) and Soysallı (1523.6 mg/kg), further indicating potential salinity challenges at these sites. Trace elements, including copper (Cu), iron (Fe), manganese (Mn), and zinc (Zn), were present in low but variable concentrations, with Zn showing the highest values at Tekir (1.25 mg/kg) and Çayırözü (1.15 mg/kg), which may support specific microbial functions such as siderophore production. Due to the lack of replicate samples per location, one-way ANOVA and Tukey-Kramer HSD tests (p < 0.01) could not detect significant differences among locations for any parameter, as within-group variance was inestimable.

### 3.2 Bacterial diversity

A total of 165 microbial isolates were collected from 22 distinct soil samples taken from the wild rhizosphere region of Mount Erciyes. Based on colony morphology and Gram staining, the isolates were identified as follows: 50 *Azotobacter* sp. (30.3%), 42 *Azospirillum* sp. (25.5%), and 73 *Bacillus* sp. (44.2%). Genomic DNA was extracted from all bacterial isolates, and PCR analysis was performed to assess the presence and genetic diversity of several plant growth-promoting genes, including *nif*, *ipdC*, *accd*, *Acpho*, *Alpho*, *phy*, and *sd*. This approach enabled the identification of functional gene variants associated with plant growth promotion across the microbial community. Soil samples collected from Mount Erciyes displayed varying physicochemical characteristics based on location, which likely influenced microbial diversity and genetic functions observed. For example, the Kıranardı and Hisarcık soils, both with clay loam textures and slightly alkaline pH (7.3–7.7), had moderate organic matter (2.45–2.91%) and nitrogen content (0.12–0.15%). These conditions are suitable for nitrogen-fixing bacteria like *Azotobacter* and *Azospirillum*, which were prevalent in these regions due to the availability of nutrients and optimal pH levels.

Samples from Koçcağız and Çaylıca, featuring loamy soils with higher organic matter (3.12% and 2.75%, respectively), provided a nutrient-rich environment, which likely supports a diverse microbial community including *Bacillus* species. The variability in soil texture, organic matter, and pH across these sampling sites underscores how soil type influences the presence and diversity of plant growth-promoting genes, including those involved in nitrogen fixation and phosphate solubilization. Such diversity in microbial genes highlights the adaptive mechanisms and potential for these soils to support sustainable agriculture in response to nutrient demands.

### 3.3 Molecular screening of plant growth-promoting genes

#### 3.3.1 Nitrogen fixation genes

**Figure 1:**
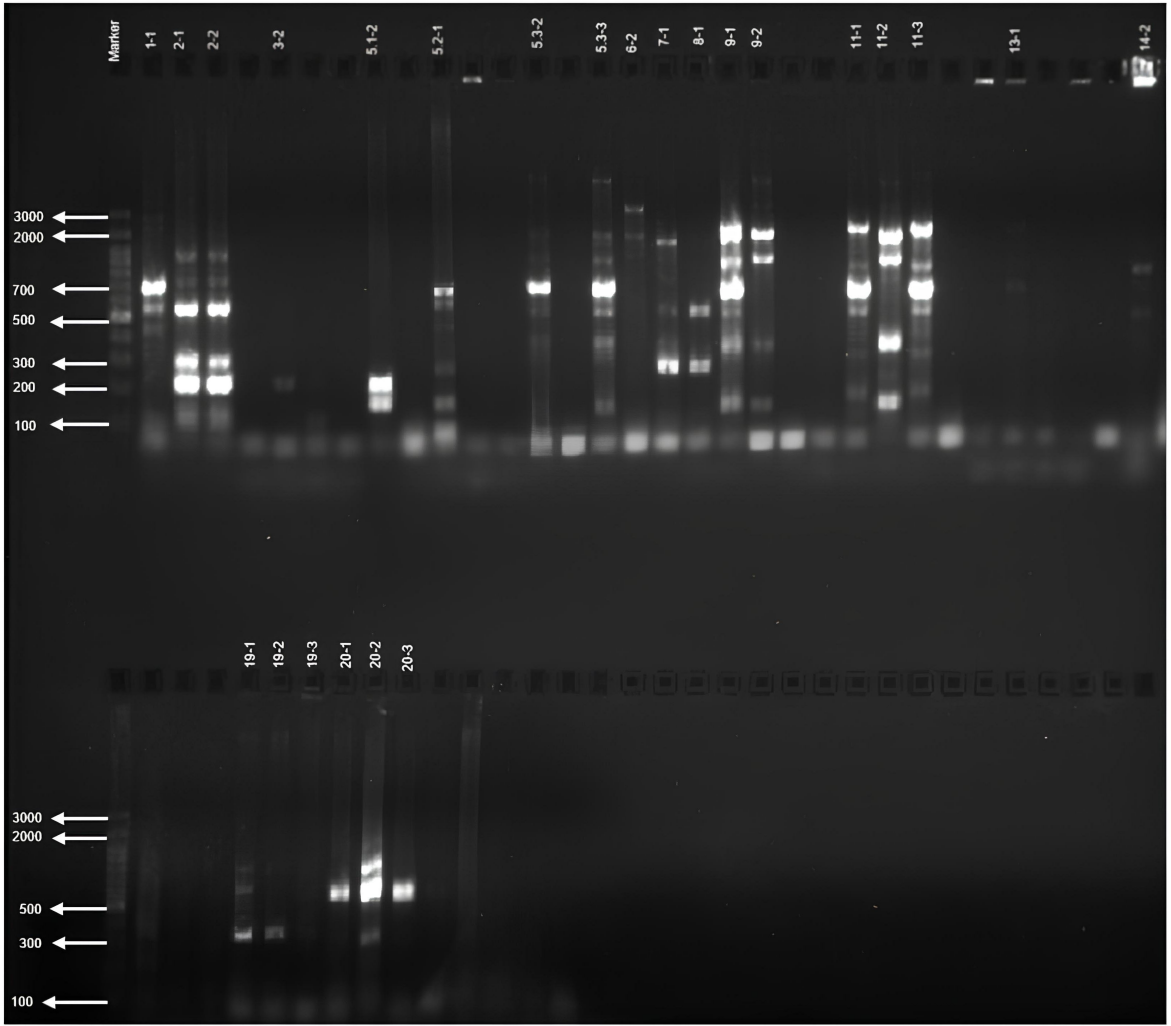
*nifH-1* gene profile of *Azotobacter* sp. isolates.

Screening of 50 *Azotobacter* sp. isolates for *nifH* genes using various primers revealed diverse amplification patterns. The *nifH-1* primer was expected to produce a 1102 bp band (**Figure 1**); however, 24 isolates exhibited bands of varying haplotypes with sizes deviating from this expectation (**Table 4**).

**Table 4:** Summary of bacterial species producing bands for plant growth-promoting genes.

Primers	*Azotobacter* sp.	*Azospirillum* sp.	*Bacillus* sp.	Band size (bp)
*nifH-1*	24	23	3	1102
*nifH-2*	–	18	16	246
*nifH-3*	–	10	2	346
*nifH-4*	8	4	–	323
*nifH-5*	4	2	2 (different size)	323
*nifH-6*	6	–	–	360
*nifH-7*	Variable haplotypes	3	–	205
*nifH-8*	–	Variable haplotypes	2	205
*nifH-9*	–	Variable haplotypes	Variable haplotypes	–
*ipdC-6*	–	21	–	Variable
*ipdC-3 to 8*	–	–	18 (500 bp larger than 200)	200
*accd*	39	11	29	800
*acpho-1*	11	8	4	734
*alpho/phy*	–	–	–	–
*sd-2/3*	–	11	7	1685

Screening with the *nifH-4* primer resulted in eight isolates producing the expected 323 bp band, while the *nifH-5* primer yielded the anticipated 323 bp band in four isolates. The *nifH-6* primer generated a 360 bp band in six isolates, consistent with expectations. In contrast, the *nifH-7* primer produced gene patterns with a range of haplotypes significantly different from the expected 205 bp size. These findings indicate considerable genetic diversity in *nifH* genes among *Azotobacter* sp. isolates from different regions (**Supplementary Figure 1**).

Screening of 42 *Azospirillum* sp. isolates with *nifH* primers also showed varied results. The *nifH-1* primer was expected to amplify a 1102 bp band, but 23 isolates displayed bands of different haplotypes with sizes deviating from this standard. The *nifH-2* primer produced the expected 246 bp band in 18 isolates, while the *nifH-3* primer yielded a 346 bp band in 10 isolates. For the *nifH-4* primer, four isolates generated the expected 323 bp band, and the *nifH-5* primer produced the anticipated 323 bp band in two isolates. The *nifH-7* primer resulted in three isolates showing the expected 205 bp band. Additionally, screening with the *nifH-8* primer revealed gene patterns with diverse haplotypes (**Supplementary Figures 2A, B**).

For 73 *Bacillus* sp. isolates, screening with *nifH* primers identified 37 isolates producing bands with various primers. The *nifH-1* primer was expected to yield a 1102 bp band, and three isolates met this expectation. The *nifH-2* primer produced the anticipated 246 bp band in 16 isolates, while the *nifH-3* primer resulted in two isolates showing the expected 346 bp band. Screening with the *nifH-5* primer revealed three isolates with bands deviating from the expected 323 bp size, though two isolates produced the anticipated 323 bp band. The *nifH-8* primer identified two isolates with the expected 205 bp band. Finally, screening with the *nifH-9* primer indicated gene patterns with diverse haplotypes (**Supplementary Figure 3**).

#### 3.3.2 Indole pyruvate decarboxylase genes

Screening of 50 *Azotobacter* sp. isolates with nine *ipdC* primers yielded no amplification products, indicating the absence of detectable *ipdC* genes in these isolates. In contrast, screening of 42 *Azospirillum* sp. isolates revealed that 21 isolates produced bands of varying sizes with the *ipdC-6* primer (**Supplementary Figure 4**), suggesting the presence of diverse *ipdC* gene haplotypes. Among 73 *Bacillus* sp. isolates, 18 were found to carry the *ipdC* gene. However, all positive isolates displayed band sizes approximately 500 bp larger than the expected 200 bp, indicating significant genetic variation from the anticipated profile (**Table 4**, **Supplementary Figure 5**).

#### 3.3.3 1-aminocyclopropane-1-carboxylate deaminase genes

Screening of 50 *Azotobacter* sp. isolates for the *accd* gene revealed that 39 isolates produced bands of approximately 800 bp, consistent with the expected size (**Supplementary Figure 6**). Similarly, among 42 *Azospirillum* sp. isolates, 11 displayed bands of the anticipated size for the *accd* gene (**Supplementary Figure 7**). For *Bacillus* sp., 29 out of 73 isolates were found to carry the *accd* gene, all producing bands of the expected size (**Table 4**, **Supplementary Figure 8**).

#### 3.3.4 Phosphate-solubilizing genes *(acpho, alpho and phy)*

Screening with the *Acpho-1* primer identified 11 *Azotobacter* sp., 8 *Azospirillum* sp., and 4 *Bacillus* sp. isolates that produced a band of the expected 734 bp size, indicating the presence of the acid phosphatase gene (**Supplementary Figures 9**-**11**). In contrast, PCR amplification using *Alpho* and *phy* primers yielded no bands for any of the tested bacterial isolates. However, some *Bacillus* sp. isolates produced non-specific bands (**Table 4**), which do not confirm the presence of these genes due to their lack of specificity.

#### 3.3.5 Siderophore biosynthesis genes

**Figure 2:**
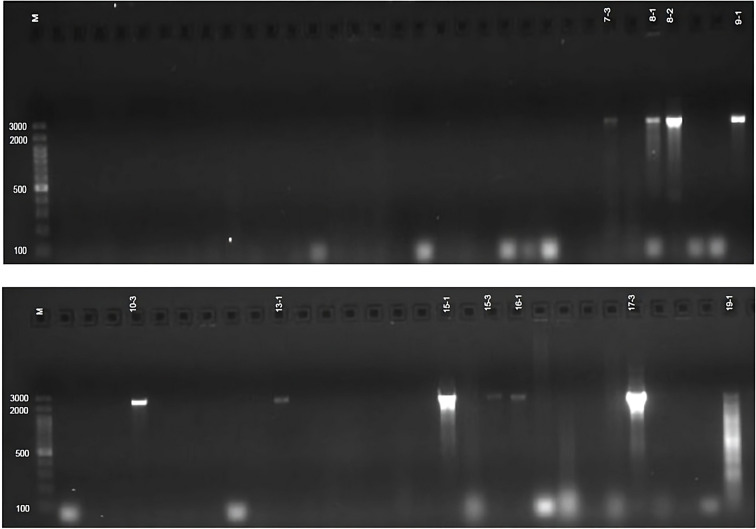
*sd-3* genes profile of *Bacillus* sp. Isolates.

PCR screening for siderophore biosynthesis (*sd*) genes using *sd-2* and *sd-3* primers revealed that none of the 50 *Azotobacter* sp. isolates carried these genes. In contrast, 11 *Azospirillum* sp. isolates and 7 *Bacillus* sp. isolates produced the expected 1685 bp band, confirming the presence of *sd* genes (**Figure 2**). Additionally, some of these isolates exhibited extra bands of significantly larger sizes (**Table 4**, **Supplementary Figures 12**, **13**), indicating potential genetic diversity in siderophore biosynthesis pathways.

## 4 Discussion

Nitrogen (N_2_), a critical component of nucleotides, proteins, and chlorophyll in plants, is one of the most essential elements for life. Despite constituting approximately 78% of the atmosphere, nitrogen is not readily available in a form usable by plants (Galloway et al., 2003). Soil is prone to nitrogen loss through processes such as denitrification, volatilization, and leaching due to nitrogen’s high reactivity and mobility. The leached reactive forms can cause environmental issues and adverse health effects (Williamson, 2011). Nitrogen fixation, the process of converting atmospheric N_2_ into ammonia (NH_3_), varies widely among bacteria and is catalyzed by the nitrogenase enzyme complex, which consists of two main components: an iron (Fe) protein encoded by the *nifH* gene and a molybdenum-iron (MoFe) protein encoded by the nifDK genes. The nifH gene is evolutionarily conserved and serves as a marker for nitrogen fixation, with primers designed from its sequence widely used to detect the genetic potential for nitrogen fixation in bacteria. While nif genes are sometimes located on chromosomes, they are often found on plasmids alongside other nitrogen-fixing genes, such as nod genes (Auman and Speake, 2001; Mehta et al., 2003; Rosch et al., 2002). In addition to encoding nitrogenase, *nif* genes regulate other enzymes involved in nitrogen fixation. Their expression is triggered by low nitrogen and oxygen levels, particularly around the root zone. Although the nif genes encoding nitrogenase are highly conserved across nitrogen-fixing bacteria, their regulation varies among diazotrophs depending on their evolutionary lineage. Approximately 20 nif genes encode the nitrogenase complex.

Transcription of nif genes is initiated under nitrogen stress, often activated by the nitrogen-sensitive nifA protein. When fixed nitrogen is scarce, *nifC*, an RNA polymerase, induces nifA expression, which in turn activates other transcription factors. In environments with sufficient reduced nitrogen or oxygen, the nifL protein suppresses nifA, halting nitrogenase production. Studies have identified nitrogenase activity in various Bacillus species isolated from rhizosphere soils, including *B. megaterium*, *B. cereus*, *B. pumilus*, *B. circulans*, *B. licheniformis*, *B. subtilis*, *B. brevis*, and *B. firmus* (Ahmad et al., 2008; Beneduzi and Passaglia, 2011; Ding et al., 2005; Sorokin et al., 2008; Xie et al., 1998). For instance, Xie et al. (1998) isolated 14 Bacillus strains capable of acetylene reduction from rice fields along the Yangtze River in China. Ahmad et al. (2008) reported high nitrogenase activity in *B. fusiformis* from Chungbuk Province, South Korea, while Sorokin et al. (2008) highlighted the nitrogenase activity of *B. alkalidiazotrophicus*, a low salt-tolerant alkaliphile from Mongolian soda soils, demonstrating the genetic diversity of nitrogenase activity in Bacillus species across different ecosystems. This nitrogen fixation capacity directly contributes to plant growth promotion by increasing the availability of nitrogen, a limiting nutrient for plant development. For example, nitrogen-fixing Bacillus species have been shown to enhance the growth of crops such as rice and wheat by improving nitrogen uptake, leading to increased biomass and grain yield (Kuan et al., 2016).

Phytohormones are vital for signaling and regulating plant growth and development. Auxins, particularly indole-3-acetic acid (IAA), are among the most studied plant growth regulators (Del Pozo et al., 2005; Figueredo et al., 2023). Bacteria associated with plants produce phytohormones that influence plant growth (Patten and Glick, 2002), plant pathology (Glickmann et al., 1998), and microbial interactions (Sergeeva et al., 2002). Tryptophan serves as a precursor for IAA biosynthesis (Kundan et al., 2015). Over 80% of rhizosphere bacteria can produce IAA, which enhances root branching, weight, size, and surface area, improving nutrient exchange in plants (Kashyap et al., 2019). Bacillus species possess the *ipdC* gene, which encodes indole pyruvate decarboxylase, a key enzyme in IAA production via the indole-3-pyruvate (IPyA) pathway (Raddadi et al., 2008). The production of IAA by Bacillus species promotes root system development, which enhances nutrient and water uptake, leading to improved plant vigor and yield. For instance, *Bacillus subtilis* inoculation has been shown to increase root biomass and grain yield in maize by up to 15% under field conditions, demonstrating its potential for agricultural applications (Kuan et al., 2016).

*Azospirillum* species, widely distributed in tropical, subtropical, and temperate soils, produce phytohormones such as IAA, gibberellins, cytokinins, and abscisic acid, promoting plant growth (Bashan et al., 2014; Creus et al., 2004). Of the 18 identified *Azospirillum* species (Baldani et al., 2014), few are known to enhance plant growth, with *A. brasilense* being the most commonly used to improve crop yields under field conditions. *A. brasilense* synthesizes IAA from tryptophan through three pathways: indole pyruvic acid (IPyA), tryptamine, and indole acetonitrile (Carreño-López et al., 2000; Spaepen et al., 2007a, 2007b). The IPyA pathway, involving transamination of tryptophan to IPyA by aromatic amino acid aminotransferases, followed by decarboxylation to indole acetaldehyde and oxidation to IAA, is the best-characterized. The *ipdC* gene, encoding phenylpyruvate decarboxylase, is critical in this pathway, and its presence has been confirmed in many *Azospirillum* strains (Jijón-Moreno et al., 2015). The application of *A. brasilense* has been associated with significant increases in crop productivity, including a 20–30% yield improvement in wheat and maize under field conditions, primarily due to enhanced root development and nutrient assimilation (Bashan et al., 2014).

1-Aminocyclopropane-1-carboxylic acid (ACC), a natural ethylene precursor, regulates seed germination, senescence, fruit ripening, wound healing, and plant development (Deikman, 1997). ACC deaminase, found in plant growth-promoting bacteria, converts ACC into α-ketobutyrate and ammonium, promoting root elongation during seed germination (Wenbo et al., 2003). Bacillus species with the *accd* gene enhance root-shoot development and increase dry and wet weights in plants (Ghosh et al., 2003; Raddadi et al., 2008). By lowering ethylene levels, ACC deaminase-producing bacteria mitigate stress responses in plants, leading to improved growth under adverse conditions such as drought or salinity. For example, *Bacillus amyloliquefaciens* has been shown to increase tomato plant biomass by 25% under salt stress, highlighting its role in enhancing plant resilience and productivity (Raddadi et al., 2008).

Although phosphorus (P) is abundant in soil, its insoluble form limits plant uptake, making it a critical constraint for growth. Soil phosphorus exists as mineral phosphates (e.g., calcium phosphates, hydroxyapatite, and rock phosphate) and organic phosphates (e.g., phosphoesters, phytates, or inositol phosphates), but plants can only absorb it as soluble monobasic or dibasic ions (Glass, 1989). Phosphate-solubilizing bacteria produce enzymes that convert insoluble phosphates into soluble forms (Nautiyal, 1999). The primary mechanism involves organic acid production, which acidifies the microbial environment, displacing Ca²^+^ ions to release ionic phosphate. Bacillus species are key phosphate-solubilizing bacteria, utilizing enzymes like acid phosphatases (Acpho), alkaline phosphatases (Alpho), and phytases (phy) for dephosphorylation (Abdallah et al., 2018; Fitriatin et al., 2014; Raddadi et al., 2008; Rodríguez and Fraga, 1999). Phosphatase genes in *B. cereus* and *B. thuringiensis* show 99% homology with those of *B. cereus* ATCC 14579T (Raddadi et al., 2008). Alkaline phosphatases, encoded by *phoA*, *phoD*, and *phoX* gene families, and phytases, with varying catalytic mechanisms, exhibit high microbial diversity (Lim et al., 2007; Mullaney and Ullah, 2003; Ragot et al., 2015; Zimmerman et al., 2013). In marine systems, *phoD* and *phoX* predominate over *phoA*, while *phoD* is the most abundant alkaline phosphatase gene in terrestrial soils, correlating with potential phosphatase activity (Fraser et al., 2015; Luo et al., 2009; Neal et al., 2018; Sebastian and Ammerman, 2009). Phosphate solubilization by Bacillus species enhances phosphorus availability, leading to improved plant growth and yield. For instance, *B. megaterium* has been reported to increase phosphorus uptake in soybean, resulting in a 10–15% increase in seed yield under greenhouse conditions (Fitriatin et al., 2014).

Iron (Fe) deficiency, an abiotic stress, can reduce crop yields (Kobayashi et al., 2005). Plants release chelators and phytosiderophores to bind Fe³^+^, enhancing its solubility and reducing it to Fe²^+^ for uptake. Rhizospheric bacteria, including Bacillus species like *B. cereus*, *B. anthracis*, and *B. thuringiensis*, produce siderophores via non-ribosomal peptide synthesis (Cendrowski et al., 2004; Chaabouni et al., 2012; Raddadi et al., 2008; Wilson et al., 2006). These bacteria carry siderophore biosynthesis genes (*sd*) and produce siderophores like bacillibactin (a catecholate-type siderophore) in Bacillus and azotobactin in *A. vinelandii* (Raddadi et al., 2008). Plants in non-sterile soils show no iron deficiency symptoms, indicating the role of microbial siderophores in enhancing iron uptake (Masalha et al., 2000). Siderophores also combat plant pathogens by competing for iron, thus supporting plant growth (Carrillo-Castaneda et al., 2005; Chaabouni et al., 2012; Vessey, 2003). The application of siderophore-producing *Bacillus* species has been shown to improve iron nutrition in crops like maize, leading to a 10% increase in chlorophyll content and a 12% increase in biomass under iron-deficient conditions (Chaabouni et al., 2012).

In summary, the multifaceted contributions of nitrogen-fixing, phytohormone-producing, ACC deaminase-active, phosphate-solubilizing, and siderophore-producing bacteria, particularly *Bacillus* and *Azospirillum* species, significantly enhance plant growth and productivity. These mechanisms collectively improve nutrient availability, stimulate root development, mitigate stress, and enhance resistance to pathogens, leading to increased crop yields and sustainable agricultural practices. For example, field studies have demonstrated that inoculation with *Bacillus* and *Azospirillum* strains can increase crop yields by 10–30% across various crops, including maize, wheat, and soybean, under diverse environmental conditions (Bashan et al., 2014; Kuan et al., 2016). These findings underscore the potential of these bacteria as biofertilizers to enhance plant production, offering a sustainable alternative to chemical fertilizers while addressing global food security challenges.

## 5 Conclusion

Plant growth-promoting rhizobacteria (PGPR) exhibit significant genetic diversity in key genes, including those for nitrogen fixation (*nif*), indole pyruvate decarboxylase (*ipdC*), 1-aminocyclopropane-1-carboxylate deaminase (*accd*), phosphate solubilization (*Acpho*, *Alpho*, *phy*), and siderophore biosynthesis (*sd*). These genes enable PGPR to fix atmospheric nitrogen, synthesize phytohormones, and enhance the availability of phosphate and iron, thereby promoting plant growth and resilience under diverse environmental conditions. This study investigates the genetic diversity of PGPR traits in bacterial isolates from Mount Erciyes, Türkiye, revealing the influence of local soil properties:

Hisarcık: Nutrient-rich, neutral to slightly alkaline soils harbor diverse nif genes, particularly in *Azotobacter* species, supporting robust nitrogen fixation in fertile environments.

Kıranardı: Soils with moderate pH and balanced micronutrients foster diversity in *accd* and phosphate-solubilizing genes, enhancing plant stress tolerance and nutrient uptake.

Kepez: Iron-deficient soils at higher altitudes show increased diversity in *sd* genes among *Bacillus* isolates, improving iron acquisition for both plants and microbes.

Endürlük: Acidic soils with variable mineral content promote diversity in phosphate-solubilizing genes, reflecting microbial adaptation to phosphorus scarcity.

The variability in PGPR traits closely corresponds to soil composition and altitude, highlighting the adaptability of rhizobacteria to specific ecological niches. These insights emphasize the potential of tailored microbial consortia to support sustainable agriculture.

## Data Availability

The datasets presented in this study can be found in online repositories. The names of the repository/repositories and accession number(s) can be found in the article/**Supplementary Material**. Further inquiries can be directed to the corresponding author.
