# Supplementary material for: Ecological and genetic diversity of plant growth-promoting genes in rhizobacteria isolated from the rhizosphere of wild flora on Mount Erciyes, Türkiye
Source: Front Plant Sci. 2025 Sep 3;16:1657785. doi: 10.3389/fpls.2025.1657785 (PMC12440964; doi:10.3389/fpls.2025.1657785)
Supplement: Supplementary file 1 [file DataSheet1.pdf]

Supplementary Material

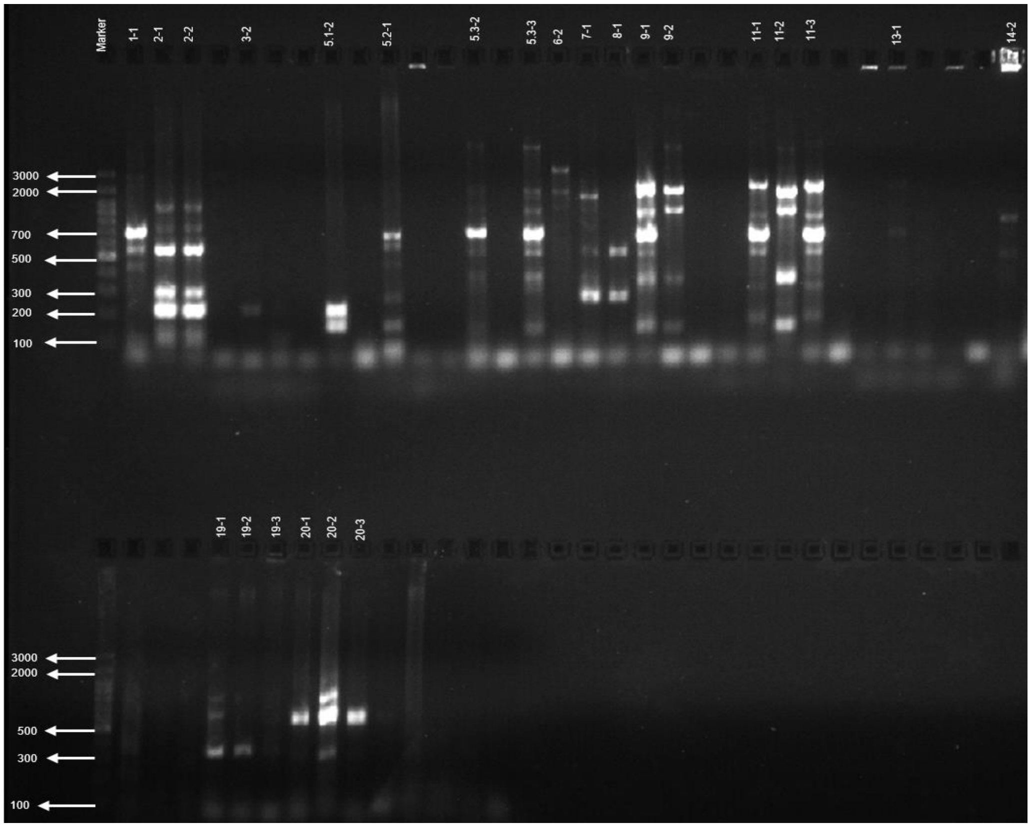

nifH-1

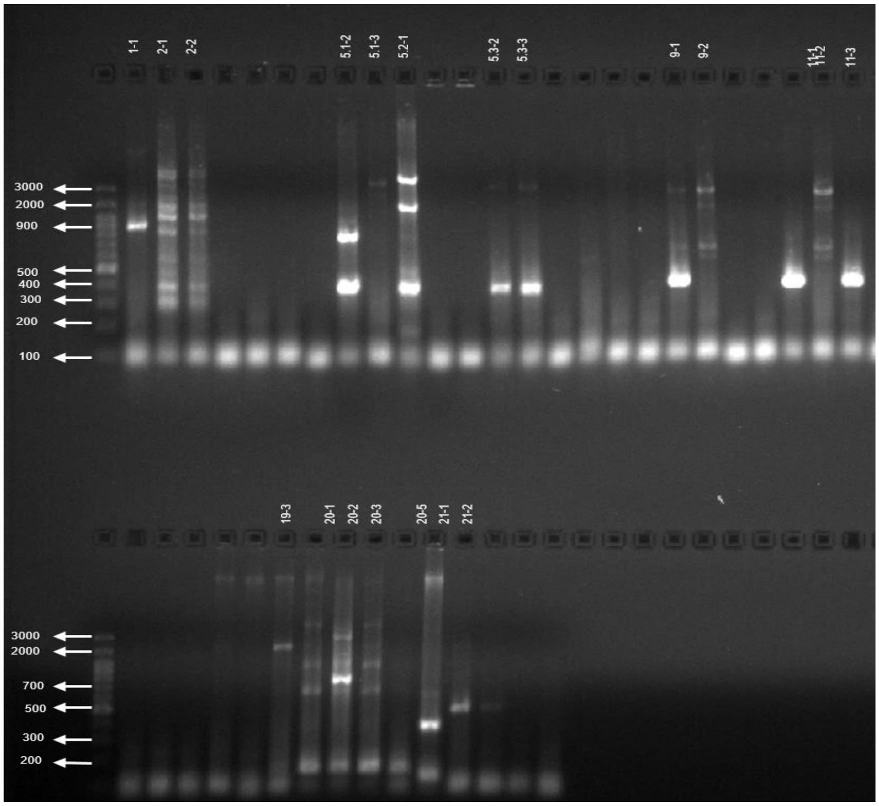

nifH-4

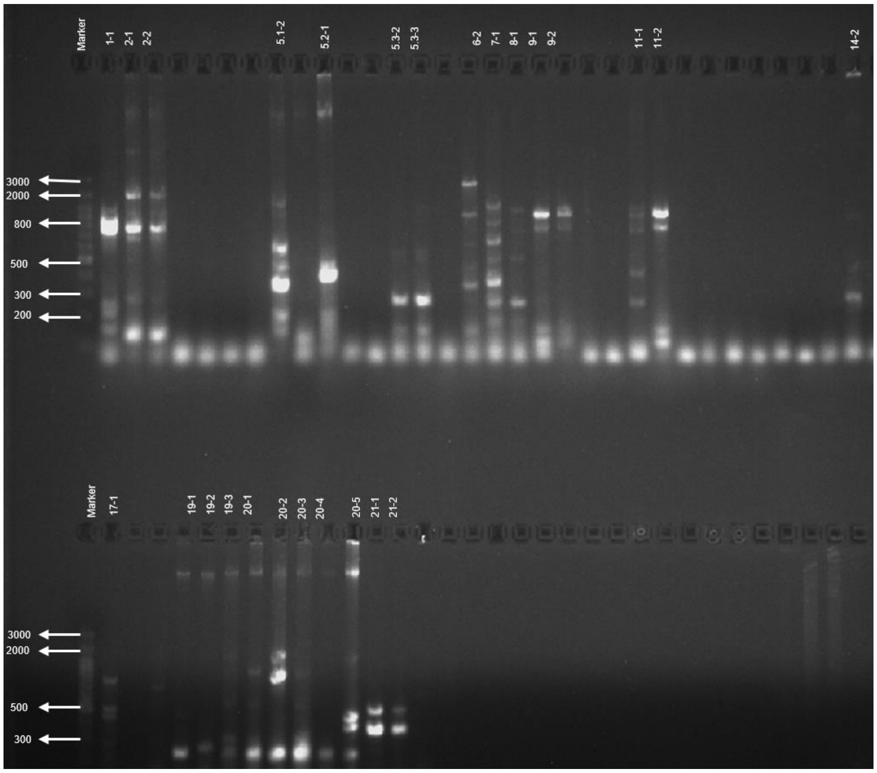

nifH-5

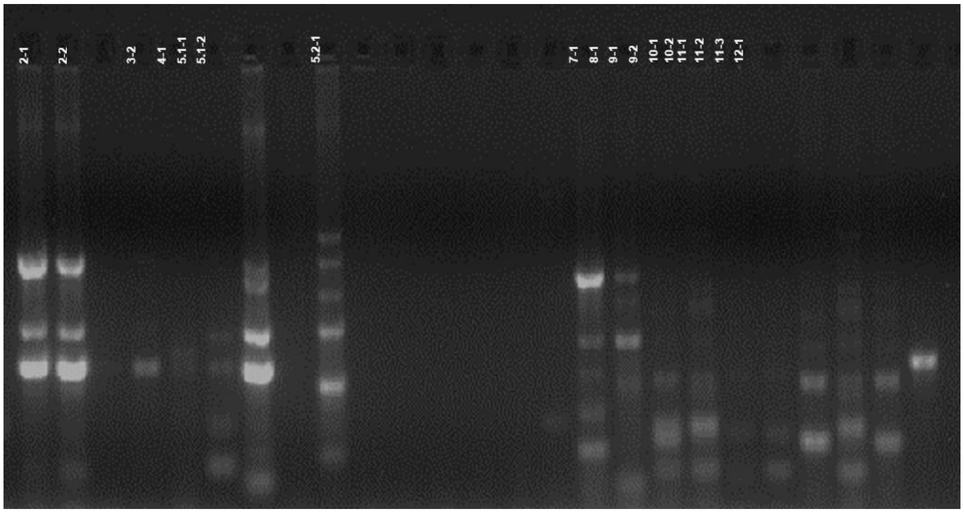
2-1
2-2
3-2
4-1
5.1-1
5.1-2
5.2-1
7-1
8-1
9-1
9-2
10-1
10-2
11-1
11-2
11-3
12-1

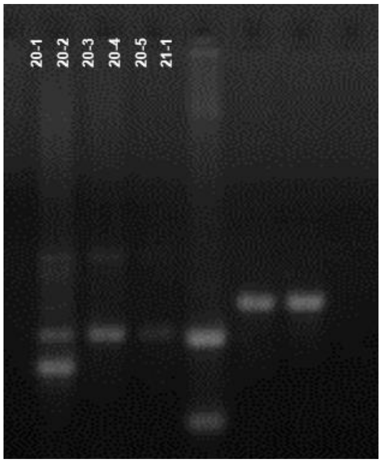
20-1
20-2
20-3
20-4
20-5
21-1

nifH-6

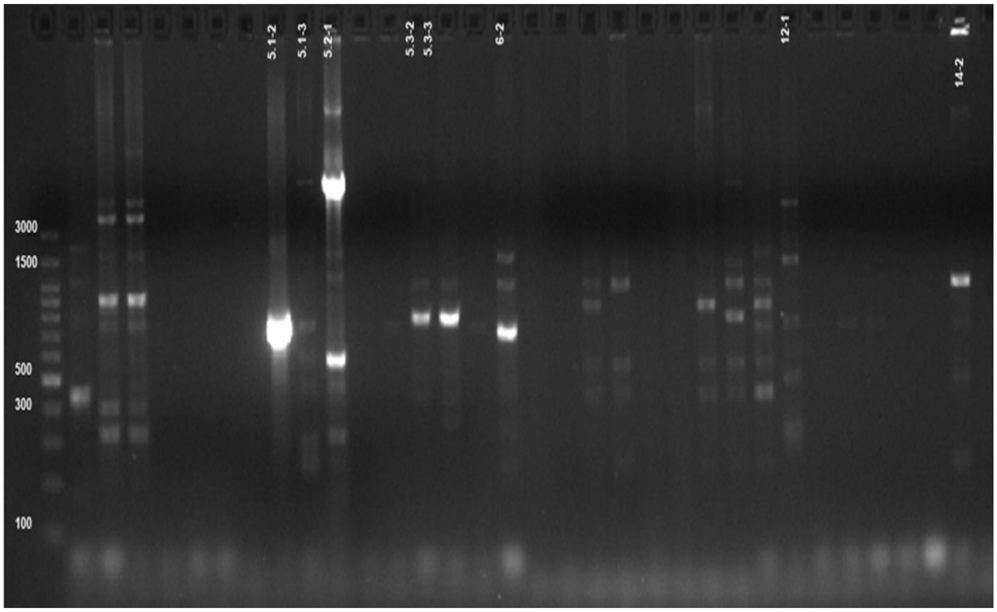
3000
1500
500
300
100
5.1-2
5.1-3
5.2-1
5.3-2
5.3-3
6-2
12-1
14-2

nifH-7

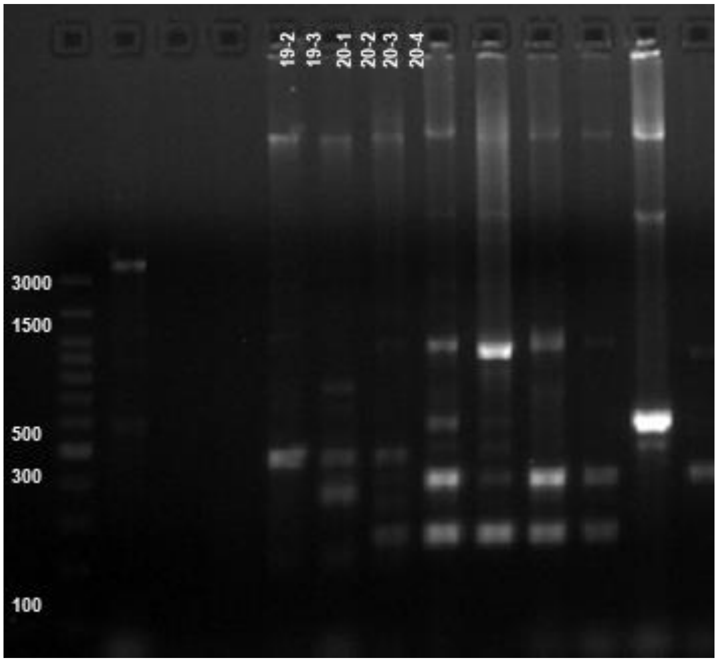

nifH-7

Supplementary Figure 1 *nifH-1,4,5,6* and *7* genes profile of *Azotobacter* sp. isolates

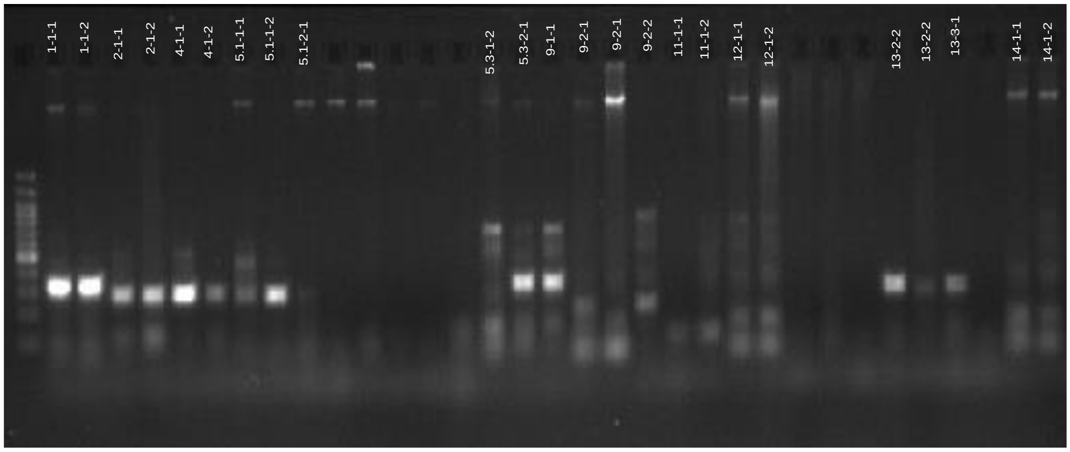

nifH-1

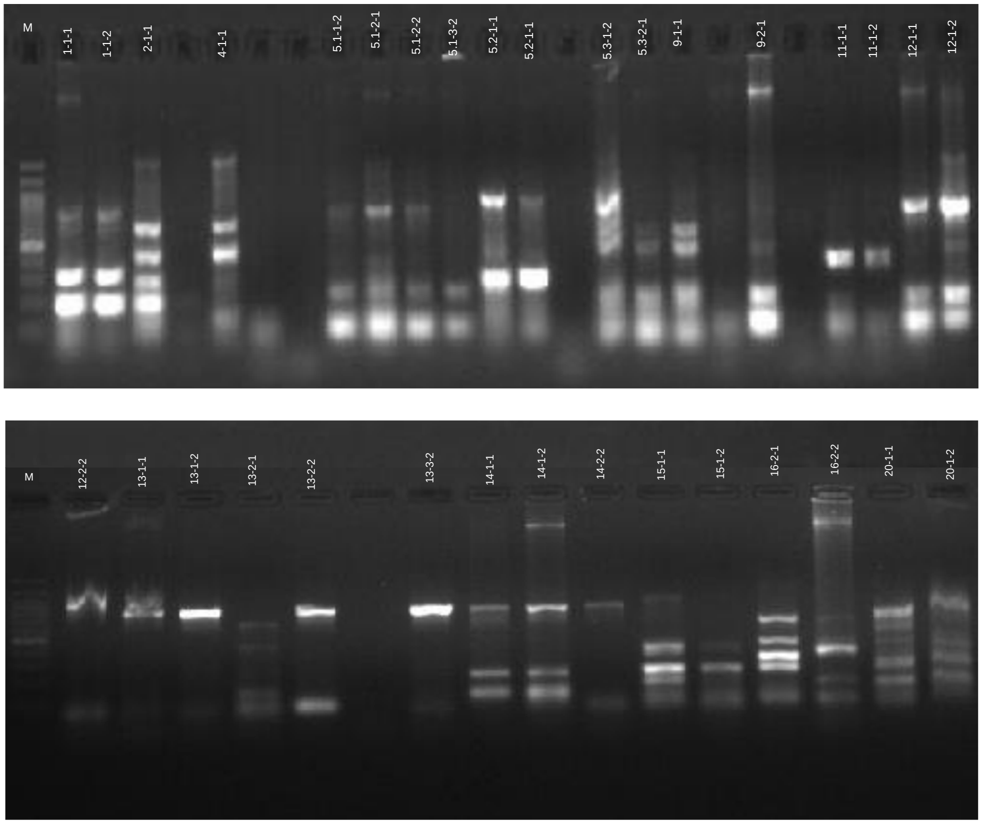
M
1-1-1
1-1-2
2-1-1
4-1-1
5.1-1-2
5.1-2-1
5.1-2-2
5.1-3-2
5.2-1-1
5.2-1-1
5.3-1-2
5.3-2-1
9-1-1
9-2-1
11-1-1
11-1-2
12-1-1
12-1-2
M
12-2-2
13-1-1
13-1-2
13-2-1
13-2-2
13-3-2
14-1-1
14-1-2
14-2-2
15-1-1
15-1-2
16-2-1
16-2-2
20-1-1
20-1-2

nifH-2

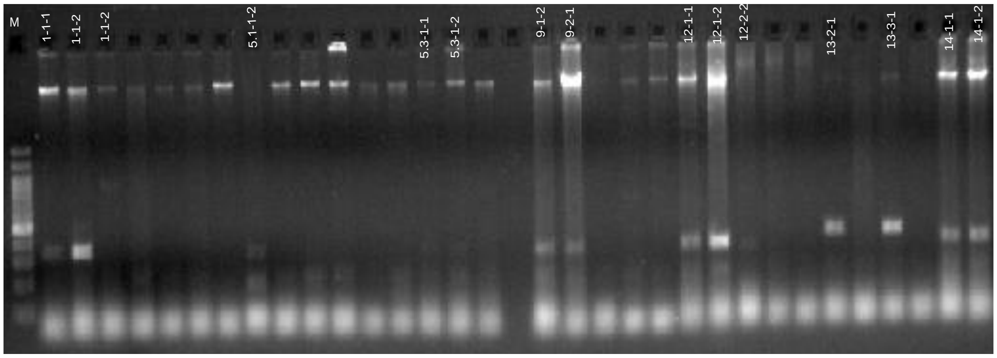
M
1-1-1
1-1-2
1-1-2
5.1-1-2
5.3-1-1
5.3-1-2
9-1-2
9-2-1
12-1-1
12-1-2
12-2-2
13-2-1
13-3-1
14-1-1
14-1-2

nifH-3

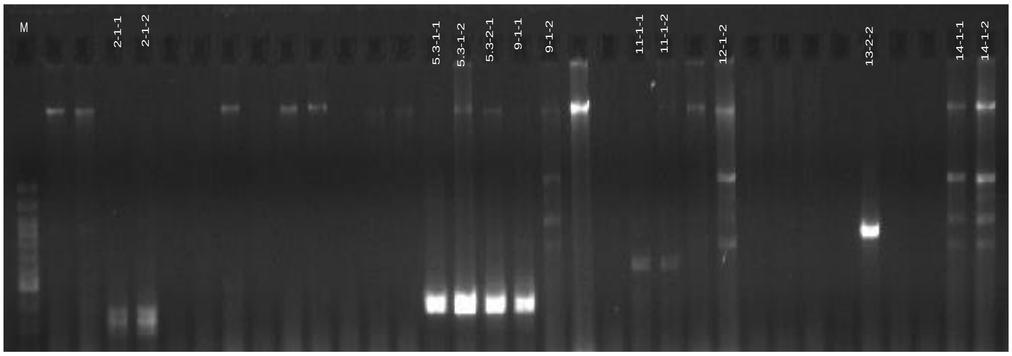

nifH-4

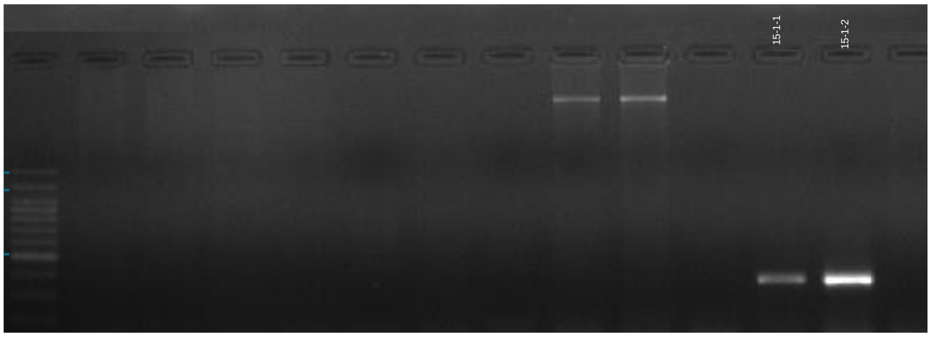

nifH-5

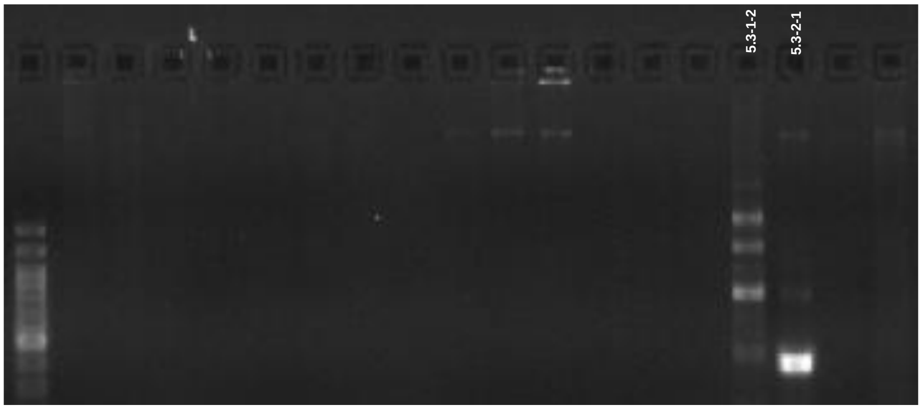

nifH-7

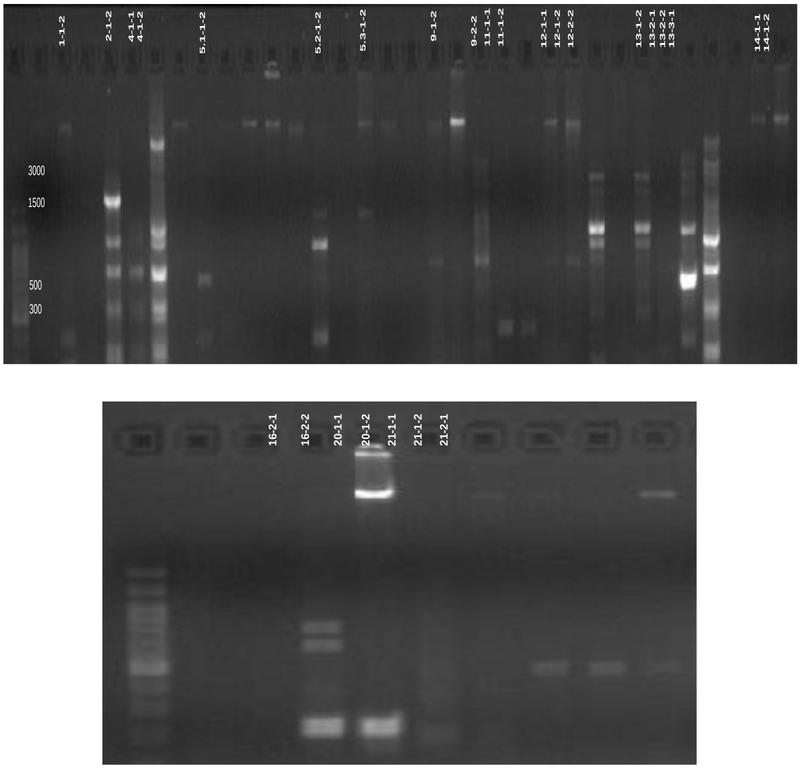

nifH-8

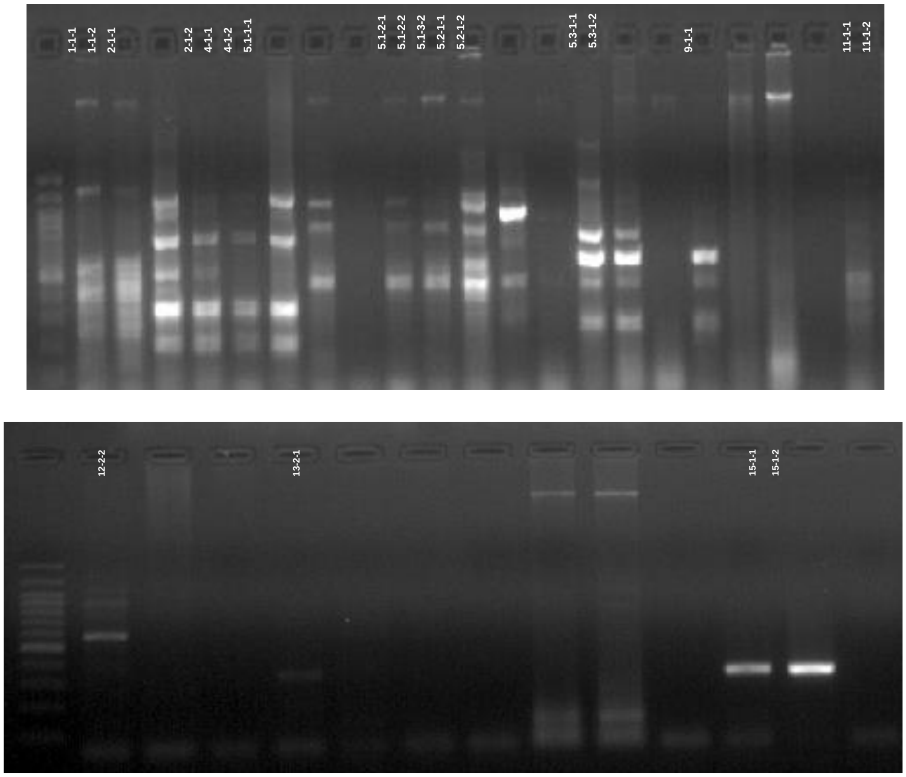

nifH-9

Supplementary Figure 2 *nifH-1,2,3,4,5,7,8* and *9* genes profile of *Azospirillum* sp. isolates

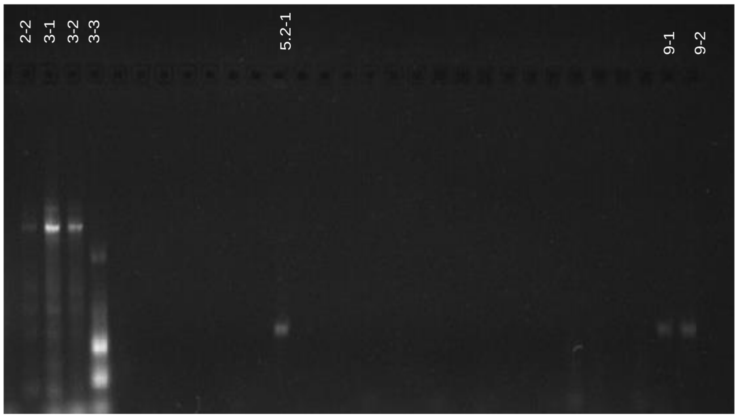

nifH-1

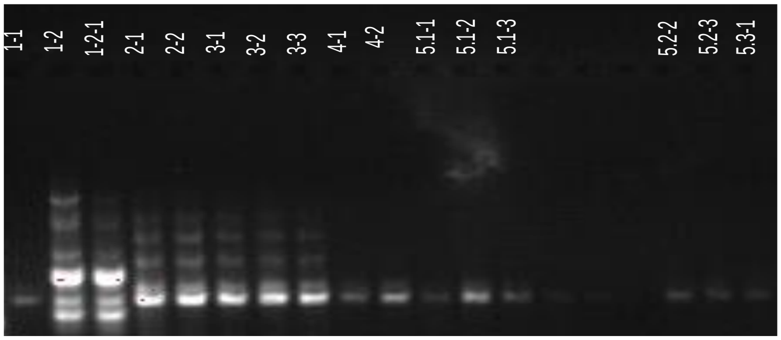

nifH-2

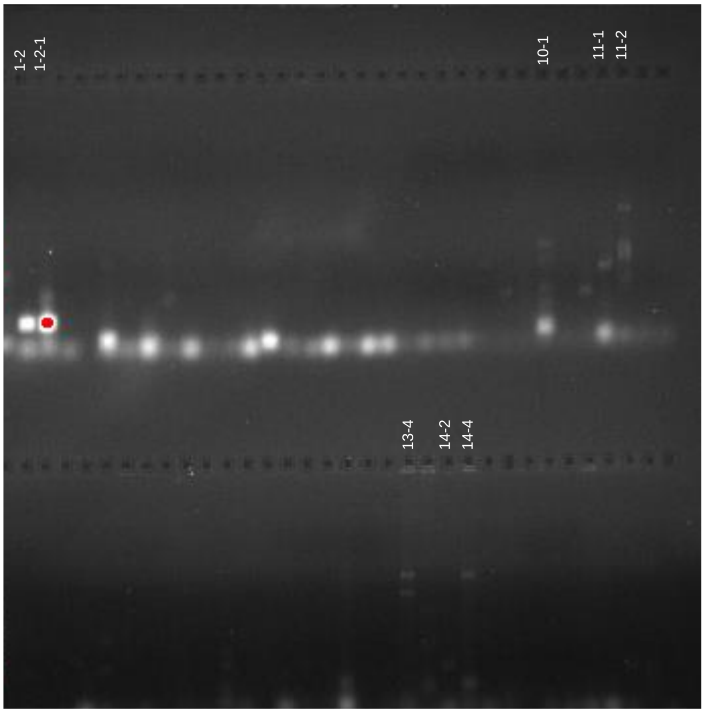

nifH-3

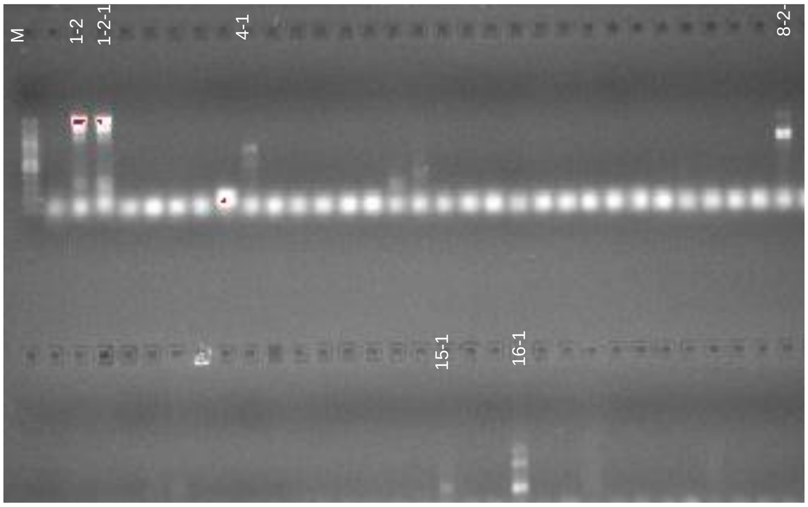

nifH-5

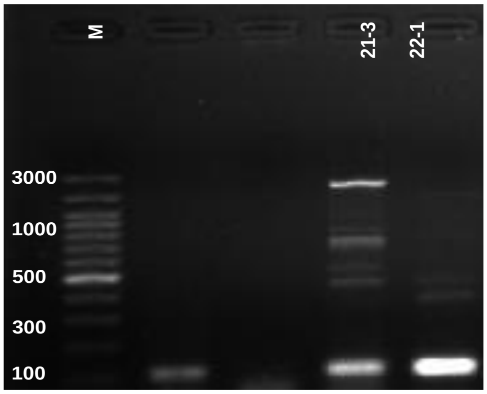

nifH-8

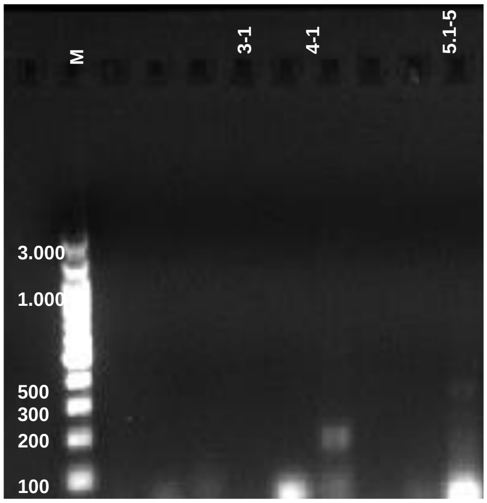

nifH-9

Supplementary Figure 3 *nifH-1,2,3,5,8* and *9* genes profile of *Bacillus* sp. isolates

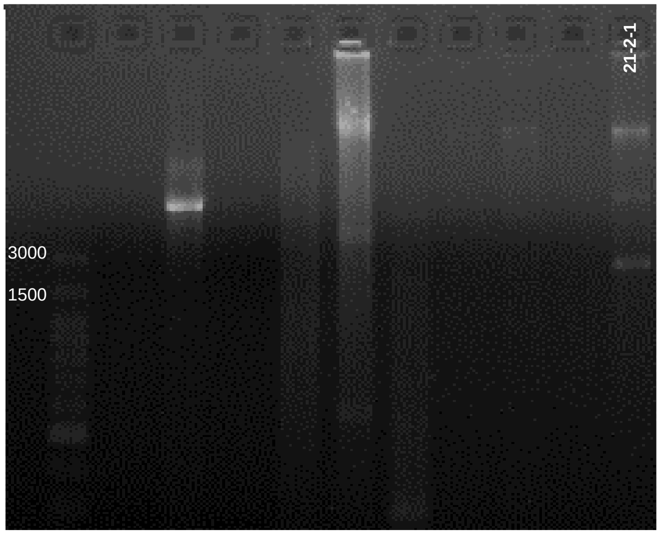

ipdC-6

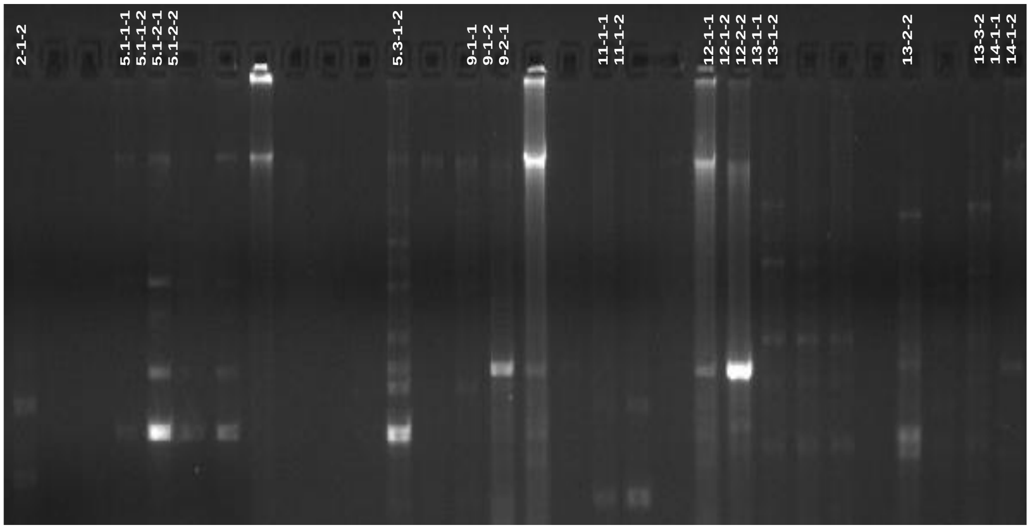

ipdC-6

Supplementary Figure 4. *ipdC-6* genes profile of *Azospirillum* sp. isolates

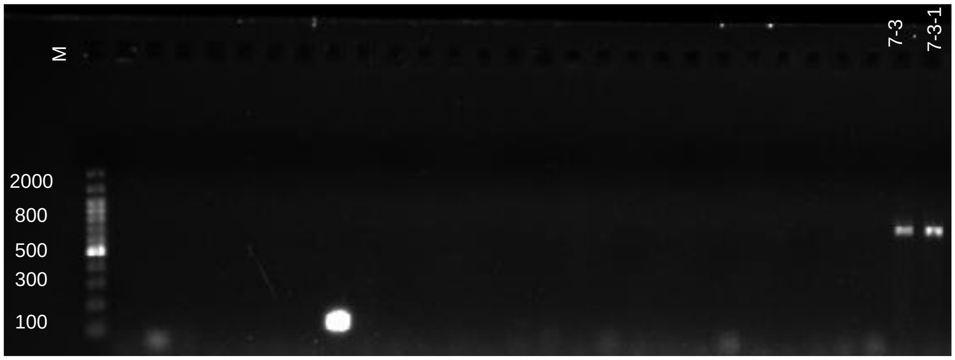

ipdC-3

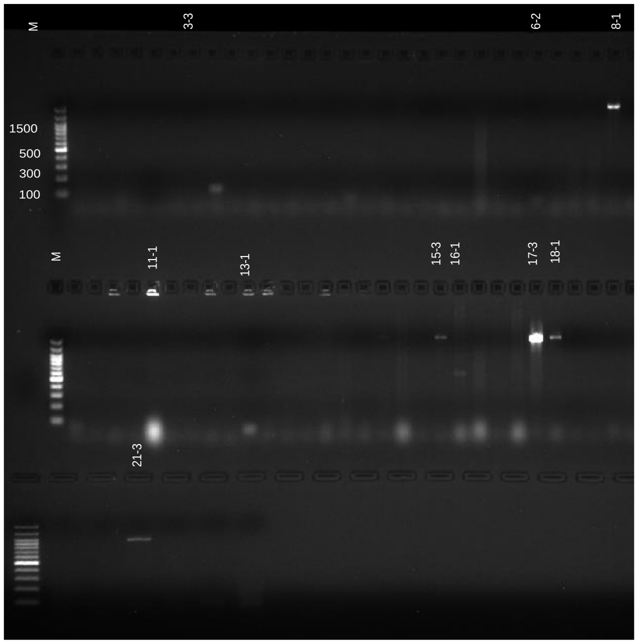

ipdC-4

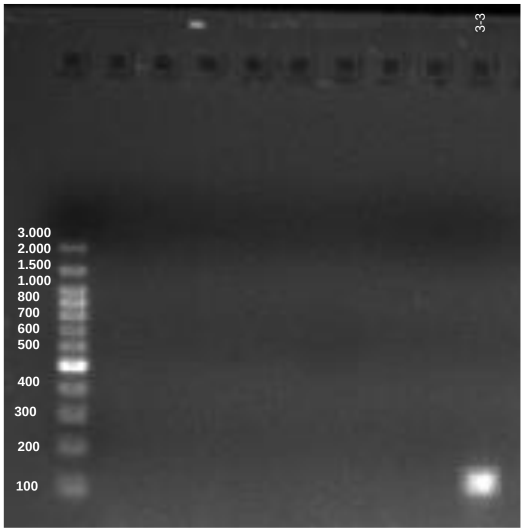

ipdC-5

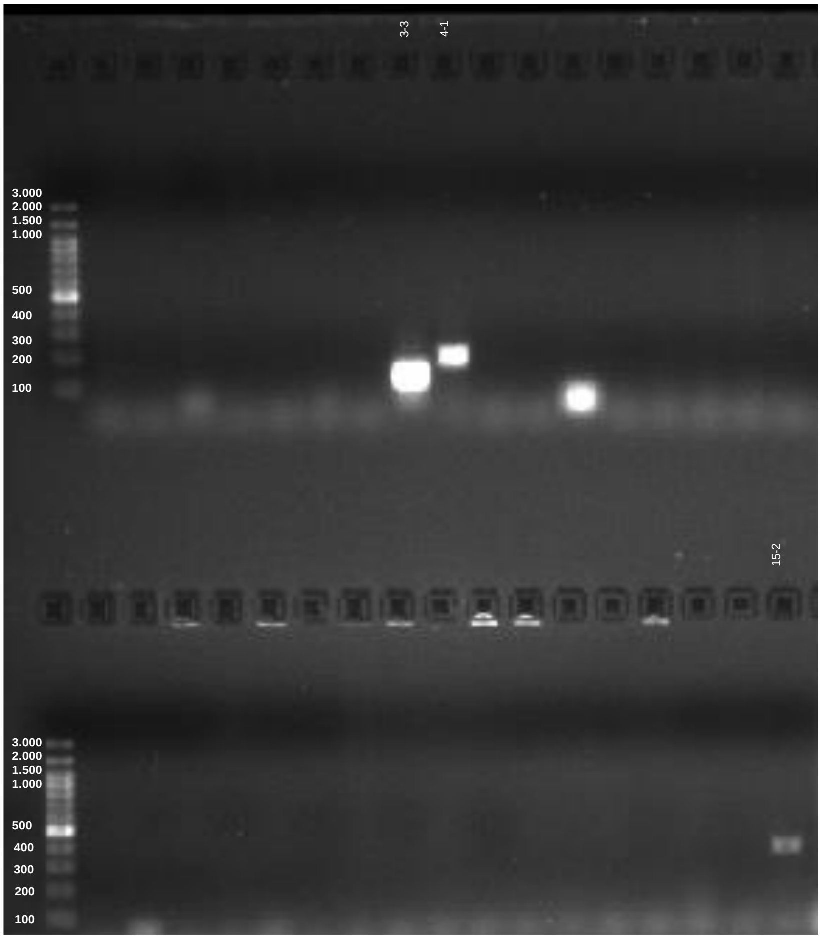

ipdC-6

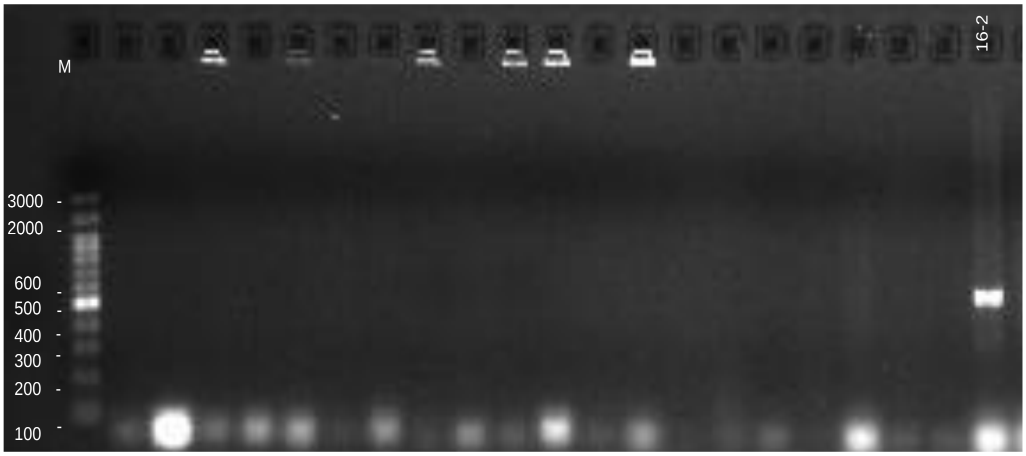

ipdC-8

Supplementary Figure 5. *ipdC-3,4,5,6* and *8* genes profile of *Bacillus* sp. isolates

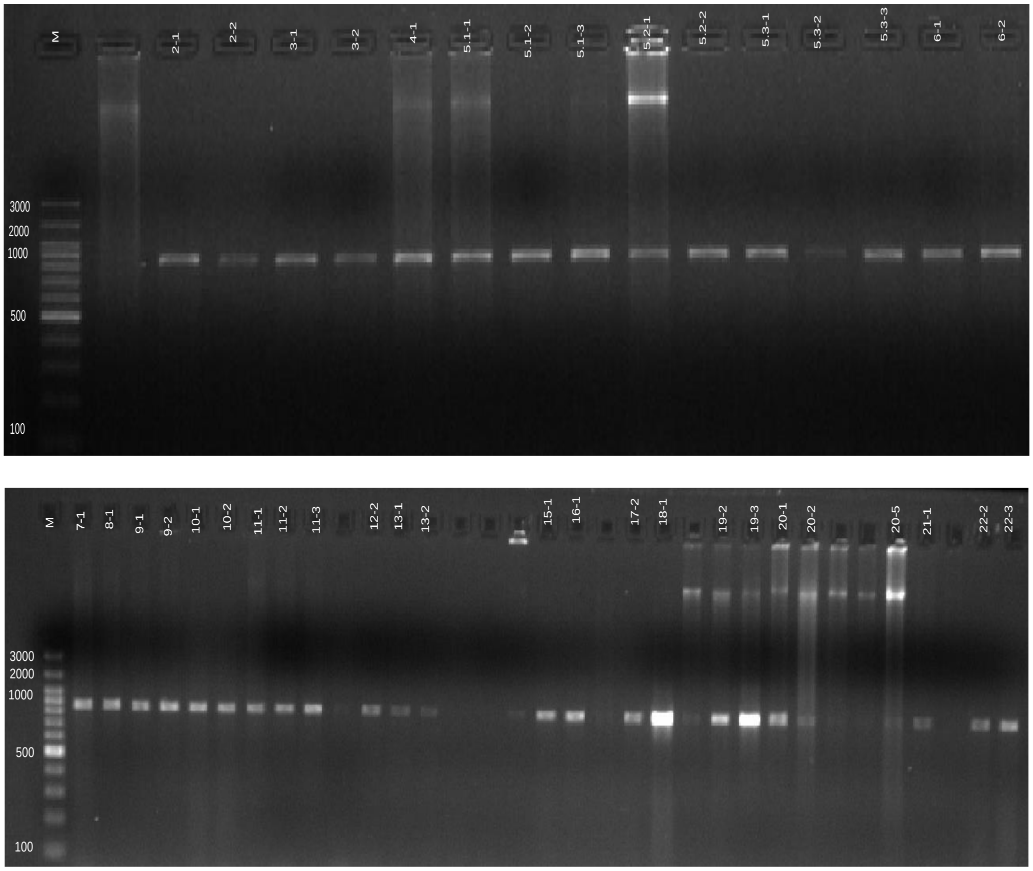

accd

Supplementary Figure 6. *accd* genes profile of *Azotobacter* sp. isolates

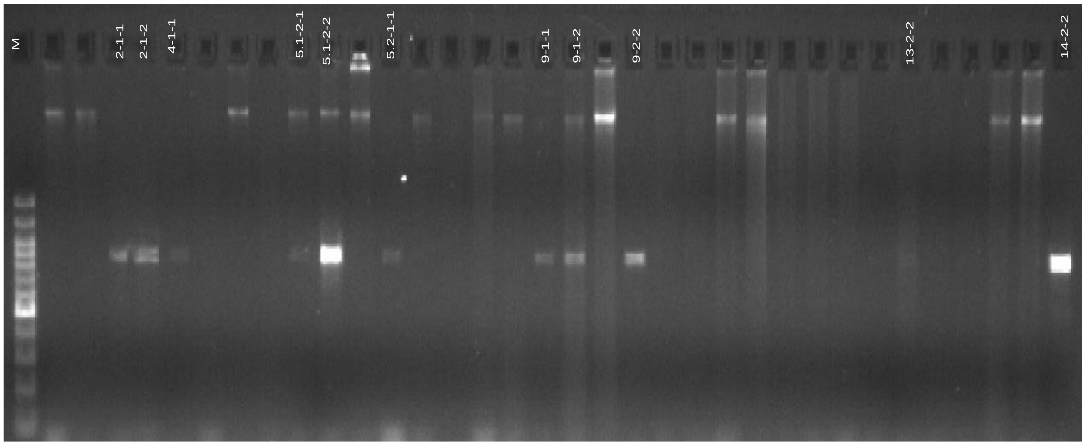

accd

Supplementary Figure 7. *accd* genes profile of *Azospirillum* sp. isolates

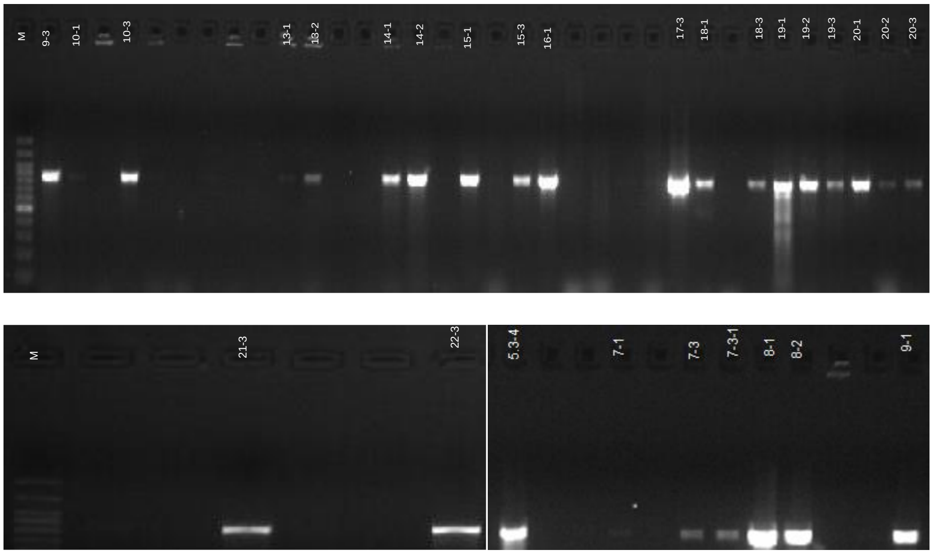

accd

Supplementary Figure 8. *accd* genes profile of *Bacillus* sp. isolates

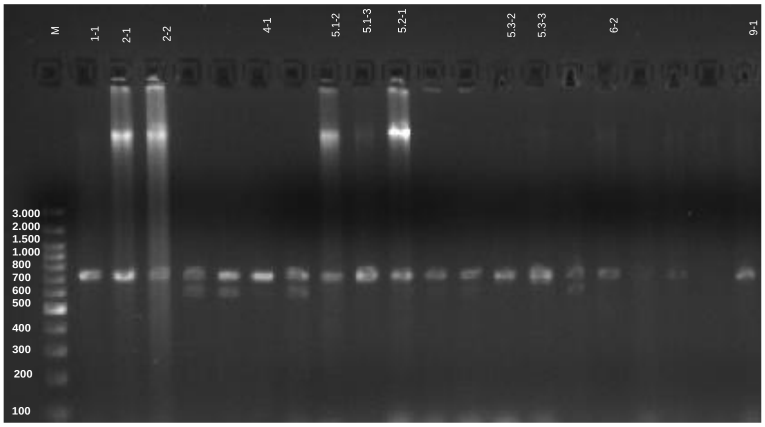

Supplementary Figure 9. *Acpho-1* genes profile of *Azotobacter* sp. isolates

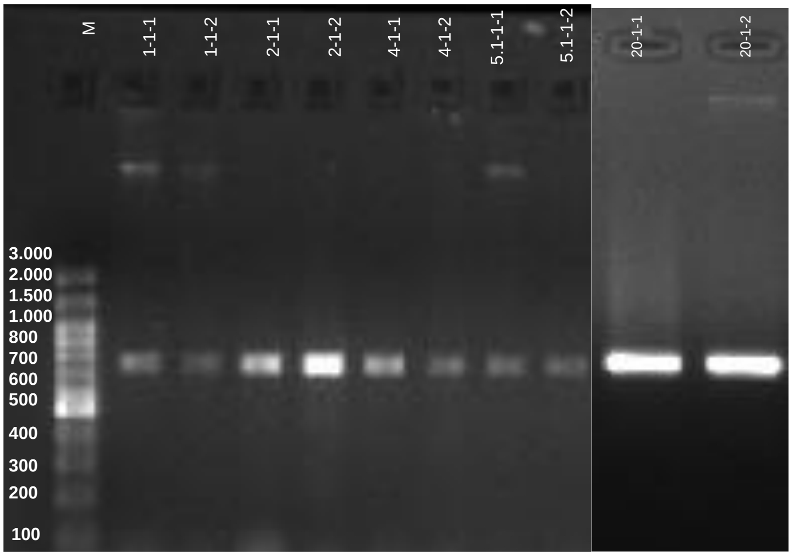

Supplementary Figure 10 *Acpho-1* and *2* genes profile of *Azospirillum* sp. isolates

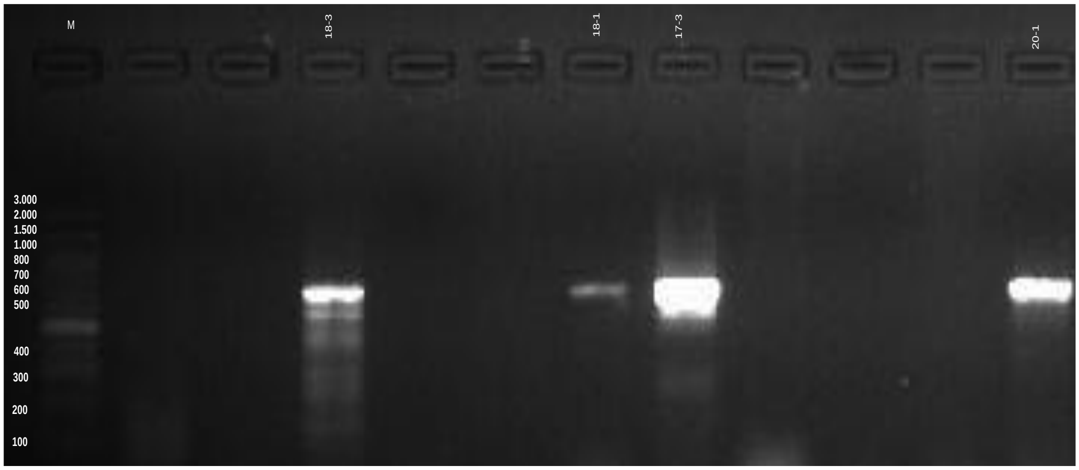

Supplementary Figure 11 *Acpho-1* genes profile of *Bacillus* sp. isolates

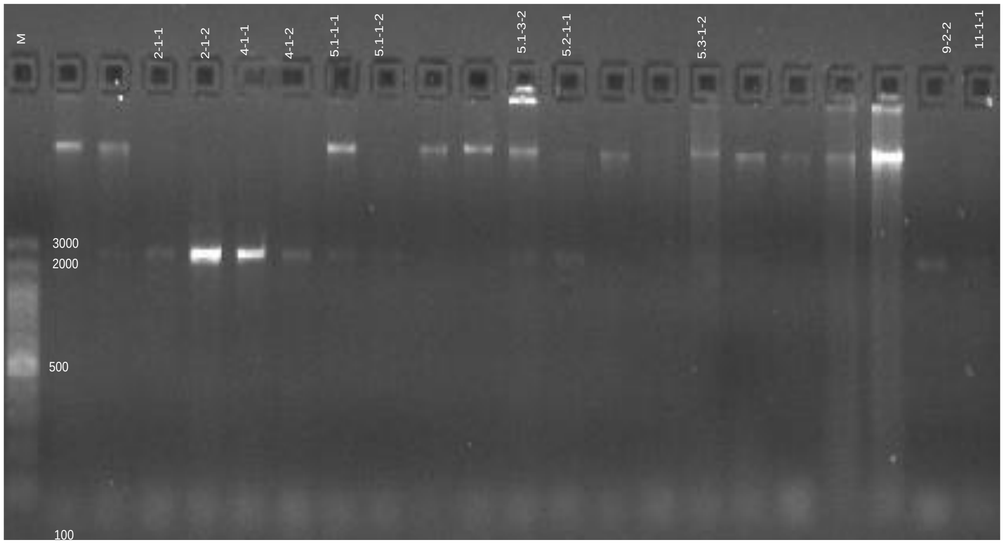

Supplementary Figure 1 *sd-2* genes profile of *Azospirillum* sp. isolates

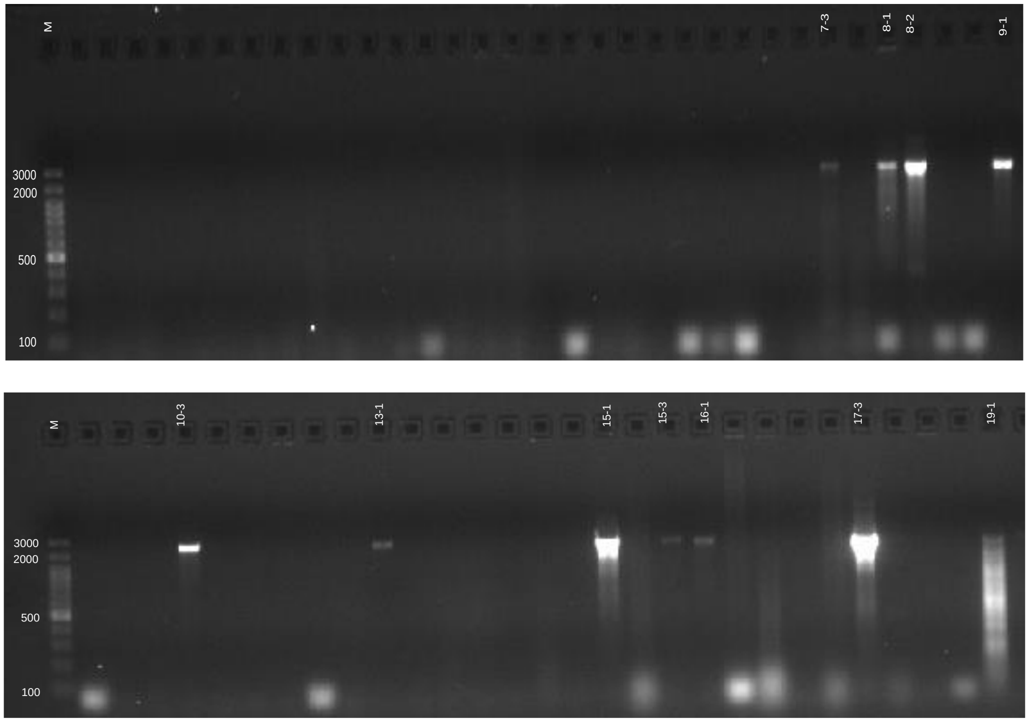

Supplementary Figure 13 *sd-3* genes profile of *Bacillus* sp. isolates
